# Methicillin resistance and antimicrobial non-susceptibility trends among *Staphylococcus aureus* clinical isolates in Aseer, Saudi Arabia (2013–2024): A retrospective laboratory-based surveillance study

**DOI:** 10.1371/journal.pone.0357789

**Published:** 2026-09-15

**Authors:** Abdulah J. Alqahtani, Yahya Shabi, Ghaday Hadi, Abdullah Algarni, Anandhalakshmi Subramanian, Ihab M. Abdelrahim, Ali Al bshabshe, Tarik Alazraqi, Safia Abdullah Mohammed, Sara Habbash, Mona M. Alfaki, Abdulah O. S. Bawazeer

**Affiliations:** 1 Department of Microbiology and Clinical Parasitology, College of Medicine, King Khalid University, Abha, Saudi Arabia; 2 Department of Medicine, Royal College of Surgeons, Dublin, Ireland; 3 Department of Family Medicine, Aseer Central Hospital, Abha, Saudi Arabia; 4 Department of Medicine, College of Medicine, King Khalid University, Abha, Saudi Arabia; 5 Department of Internal Medicine, Aseer Central Hospital, Abha, Saudi Arabia; 6 Department of Microbiology, Aseer Central Hospital, Abha, Saudi Arabia; The University of Lahore, PAKISTAN

## Abstract

**Background:**

*Staphylococcus aureus* is a leading cause of infection, and methicillin-resistant *S. aureus* (MRSA) is a high-priority resistance threat. MRSA and methicillin-susceptible *S. aureus* (MSSA) differ in non-β-lactam resistance, yet longitudinal data from Aseer, Saudi Arabia are limited. We characterized methicillin-resistance and non-susceptibility trends to inform local stewardship.

**Methods:**

We conducted a retrospective laboratory-based surveillance study of 4,194 *S. aureus* isolates from 3,122 patients (aged ≥12 years) at a tertiary-care center in Aseer, Saudi Arabia (January 2013–June 2024). Isolates were classified as MRSA or MSSA by oxacillin/cefoxitin testing to CLSI standards. We estimated annual non-susceptibility proportions and their temporal trends, compared non-susceptibility between MRSA and MSSA, and identified independent predictors of MRSA in a multivariable model accounting for repeated isolates from the same patient.

**Results:**

MRSA accounted for 2,231 isolates (53.2%; 95% CI 51.7–54.7) and MSSA for 1,963 (46.8%); most isolates were from inpatients, including 1,975/2,231 MRSA isolates (88.5%) and 1,648/1,963 MSSA isolates (84.0%). The MRSA share rose from 47.4% (2013) to 60.4% (2024), each later year being independently associated with MRSA (adjusted OR 1.09/year, 95% CI 1.06–1.12). MRSA was significantly more non-susceptible than MSSA to penicillin, ciprofloxacin, levofloxacin, erythromycin, clindamycin, gentamicin, trimethoprim–sulfamethoxazole, tetracycline, fusidic acid and rifampin (all FDR-adjusted p < 0.05), with the widest gap for fusidic acid (62.7% vs 25.0%); fusidic-acid and fluoroquinolone non-susceptibility also increased over time. No vancomycin or daptomycin non-susceptibility was detected; teicoplanin non-susceptibility was low but significantly higher in MRSA (2.8% vs 1.5%), and low-level linezolid non-susceptibility (1.5% vs 1.3%) was not confirmatory-tested.

**Conclusions:**

In this single-center Aseer surveillance, MRSA became the majority of *S. aureus* during 2013–2024 and showed broad multidrug non-susceptibility, while vancomycin and daptomycin retained full in vitro activity. These local findings support sustained laboratory-based surveillance, Aseer-tailored empirical and stewardship guidance, and confirmatory testing of the emerging low-level linezolid non-susceptibility signal.

## 1. Introduction

*Staphylococcus aureus* is a Gram-positive coccus that asymptomatically colonises the anterior nares and skin of a substantial proportion of healthy people; longitudinal studies describe persistent carriers, intermittent carriers, and non-carriers rather than a single fixed proportion colonised at any moment [1,2]. This carriage is an important reservoir and increases the risk of subsequent endogenous infection when cutaneous or mucosal barriers are breached or host immunity is impaired [3,4]. *S. aureus* causes a broad spectrum of disease, from skin and soft-tissue infections to invasive syndromes such as bacteraemia, pneumonia, osteomyelitis, septic arthritis, and infective endocarditis [2]. Its capacity to cause disease (pathogenicity) is the net result of numerous virulence determinants, including surface adhesins, immune-evasion molecules (for example protein A and coagulase), secreted cytotoxins, and biofilm formation on prosthetic material, that mediate adhesion, tissue invasion, and persistence [2,4].

Management of *S. aureus* infection is complicated by methicillin resistance, which arises principally through acquisition of the mecA gene (or its homologue mecC) carried on the mobile staphylococcal cassette chromosome mec (SCCmec); mecA encodes an alternative penicillin-binding protein, PBP2a, with low affinity for β-lactams, conferring resistance to almost all β-lactam agents [5]. Methicillin-resistant *S. aureus* (MRSA) occurs in both healthcare-associated (HA-MRSA) and community-associated (CA-MRSA) forms that differ in genetic background and epidemiology [6], although these forms increasingly overlap and cannot be distinguished by routine phenotypic testing without molecular typing. The clinical and public-health importance of MRSA reflects more than this single resistance mechanism, however: it stems from the combination of β-lactam resistance, frequent co-resistance to other antibiotic classes, and the organism’s virulence and transmissibility, which together restrict treatment options and worsen outcomes. Reflecting this burden, the World Health Organization lists MRSA among its high-priority antibiotic-resistant pathogens [7], and MRSA was estimated to be associated with more than 100,000 deaths attributable to antimicrobial resistance globally in 2019 [8].

Beyond β-lactams, MRSA isolates frequently show higher non-susceptibility than methicillin-susceptible *S. aureus* (MSSA) to several other classes, including macrolides, lincosamides, aminoglycosides, and fluoroquinolones, partly because the mobile elements carrying mecA may also carry additional resistance determinants and because resistant lineages accumulate further mutations [9,10]. These non-β-lactam resistances are mediated by diverse mechanisms, including enzymatic drug modification (for example aminoglycoside-modifying enzymes), ribosomal target modification (erm-mediated macrolide–lincosamide resistance), target-site mutation (fluoroquinolones), and active efflux [10]. MSSA nevertheless remains a major cause of serious infection and is generally treated with β-lactams, so distinguishing MRSA from MSSA and monitoring the susceptibility of both is clinically important. In Saudi Arabia, the reported proportion of *S. aureus* that is MRSA varies widely by region and setting: studies across Saudi centers have reported MRSA prevalence of roughly 9–17%, and community-associated MRSA has been reported in about 23–42% of isolates in some regions [11–13].

Although MRSA surveillance data exist in Saudi Arabia, including multicenter and regional reports, longitudinal data that simultaneously track resistance in both MRSA and MSSA and across inpatient and outpatient settings in the Aseer region of the south-west remain limited [12]. Local, longitudinal susceptibility data are needed because the relative contribution of MRSA and the pattern of resistance differ between regions and over time, and because empirical-therapy and stewardship decisions are best guided by local evidence.

Saudi healthcare facilities already operate antimicrobial stewardship programs and national treatment guidance [14]; the aim of this study is therefore not to devise empirical guidance *de novo* but to provide updated, locally specific evidence to support and refine it. Accordingly, at a tertiary-care center serving the Aseer region we conducted a retrospective laboratory-based surveillance study (January 2013–June 2024) to (i) quantify the proportion of *S. aureus* that is MRSA and its temporal trend, (ii) compare antimicrobial non-susceptibility between MRSA and MSSA, and (iii) characterize non-susceptibility trends over time, including any emerging signals against last-resort agents, to inform local empirical-therapy and stewardship decisions.

## 2. Methods

### 2.1. Study design and population

This retrospective laboratory-based surveillance study analyzed 4,194 *Staphylococcus aureus* clinical isolates obtained at a tertiary-care center serving the Aseer region of south-western Saudi Arabia between January 2013 and June 2024. The laboratory information system export used for the final analysis contained 4,194 S. aureus isolates from 3,122 unique patients aged ≥12 years. No records from patients aged <12 years were present in the final analytic dataset, and therefore no pediatric or neonatal isolates were excluded during data cleaning. From 2016 onward, the hospital did not routinely provide care for patients younger than 12 years, which explains the absence of pediatric and neonatal isolates in the later surveillance period. For consistency and to avoid implying all-age surveillance, the target population of this study is reported as patients aged ≥12 years.

Isolates were classified by methicillin-resistance status (MRSA versus MSSA) and patient-care setting (inpatient versus outpatient). All qualifying isolates were retained without de-duplication; because some patients contributed more than one isolate, within-patient correlation was accounted for in the analysis (see Statistical Analysis). The study was approved by the Aseer Institutional Review Board, Aseer Branch, Ministry of Health, Saudi Arabia (approval F7-2–2025, dated 16 April 2025), which granted a waiver of informed consent for this retrospective analysis of routinely collected, de-identified laboratory data. The medical record number was used only to link repeated isolates from the same patient and was removed before analysis; no other identifying information was accessed by the research team.

### 2.2. Microbiological methods

Bacterial identification and antimicrobial susceptibility testing were performed using validated automated systems: the BD Phoenix 100 (Becton Dickinson, USA) and the VITEK 2 (bioMérieux, France). There was no single transition date between platforms; both systems were used in parallel and interchangeably according to platform availability during routine laboratory service. Both systems were operated according to manufacturer instructions, institutional quality-control procedures, with susceptibility results interpreted according to the contemporaneous Clinical and Laboratory Standards Institute (CLSI) breakpoints in use at the time of testing; CLSI M100 editions in use spanned the 23rd through 34th editions over the study period (2013–2024) [15]. Methicillin-resistance status was determined using cefoxitin and oxacillin testing, the CLSI-recommended approach for MRSA detection in routine clinical microbiology; oxacillin/cefoxitin results therefore defined the MRSA and MSSA groups. Confirmatory testing for linezolid was not performed. Molecular confirmation was not available throughout the full 2013–2024 study period because the Xpert MRSA NxG assay was introduced later in the laboratory. After assay implementation, 100 phenotypically defined MRSA isolates from the final two surveillance years were randomly selected for confirmatory testing using the Xpert MRSA NxG assay on the GeneXpert system (Cepheid, USA). All 100 isolates were from inpatients, and all were confirmed as MRSA. The assay detects MRSA-associated targets, including mecA and the SCCmec–orfX junction. This molecular subset was used as a confirmatory quality-assurance subset and was not intended for clonal typing, SCCmec typing, or resistance-gene epidemiology across the full study period. Specimens were grouped using the predefined Source Category variable as blood, respiratory, skin/soft tissue/wound, urine/genitourinary, sterile body fluids, bone/tissue biopsy, surveillance/screening swab, line/catheter tip, eye/ear/ENT, and unspecified/other. Isolates were recovered from inpatient and outpatient settings.

### 2.3. Statistical analysis

**Study design and population.** We conducted a retrospective, laboratory-based surveillance study of *Staphylococcus aureus* clinical isolates collected at a tertiary-care center in Saudi Arabia between 2013 and 2024. The analytic cohort included *S. aureus* isolates from patients aged ≥12 years with an available year of isolation and methicillin-resistance status. No additional exclusions were applied unless essential analytic variables were missing. Because the source dataset contained no records for patients aged <12 years, no pediatric or neonatal records were removed.

**Organism classification.** Isolates were classified as methicillin-resistant *S. aureus* (MRSA) or methicillin-susceptible *S. aureus* (MSSA) using the laboratory methicillin-resistance determination (oxacillin non-susceptible versus susceptible). Oxacillin therefore defines the groups and was not analyzed as an outcome antibiotic.

**Unit of analysis.** All qualifying *S. aureus* isolates were analyzed without de-duplication; the isolate was the unit of analysis. Because some patients contributed more than one isolate, within-patient correlation was accounted for in the regression models using generalized estimating equations (GEE) with an exchangeable working correlation clustered by medical record number (MRN). Two de-duplicated cohorts were examined as sensitivity analyses: the first isolate per patient per calendar year, and the first isolate per patient across the entire study period.

**Antimicrobial susceptibility.** Susceptibility was recorded as susceptible (S) or non-susceptible (NS); in this dataset intermediate and resistant results were captured combined as a single non-susceptible code and could not be separated, so antibiotic-specific findings are reported as non-susceptibility (intermediate or resistant). Not-reported, not-tested, missing, and blank results were excluded from each antibiotic-specific denominator and were never counted as resistant; the tested denominator is reported for every antibiotic. Agents with no observed non-susceptibility (vancomycin, daptomycin) are reported descriptively.

**Descriptive and comparative statistics.** Continuous variables are summarized as median (interquartile range [IQR]) and compared with the Mann–Whitney U test; categorical variables are summarized as counts (percentages) and compared between MRSA and MSSA with the Pearson chi-square test, or the Fisher exact test when any expected cell count was < 5. These two-group comparisons treated the isolate as the unit of analysis. Standardized mean differences (SMD) were computed for baseline characteristics. Exact binomial 95% confidence intervals (CI) for annual proportions were estimated with the Wilson score method.

**Trend analysis.** Temporal trends in antimicrobial non-susceptibility were assessed for each clinically relevant antibiotic, computed separately for all *S. aureus* and within the MRSA and MSSA strata. For each series, the annual non-susceptibility proportion (2013–2024) was modeled by segmented log-linear (joinpoint) regression weighted by the inverse variance of the log-proportion, with a Haldane continuity correction for years with zero non-susceptibility, yielding the annual percent change (APC) with 95% CI. Annual estimates were restricted to years with at least 20 tested isolates in a group. A maximum of one joinpoint was permitted, tested by grid search with an F-test for improvement in fit; where a joinpoint was identified the average APC (AAPC) and segment-specific APCs were derived. Formal NCI Joinpoint Regression software was not used; a reproducible segmented-regression implementation was applied instead. Series with no non-susceptibility (e.g., vancomycin, daptomycin) or with sparse data were reported descriptively and labeled not estimable.

**Multivariable model and covariates.** The multivariable model for MRSA versus MSSA was a GEE logistic regression (all isolates; exchangeable working correlation; clustered by MRN) adjusted for calendar year, age group, sex, setting (inpatient [INP] versus outpatient [OPD]), ward category, source category, and diagnosis category, using the pre-defined categorized variables rather than raw free-text fields, and reporting the adjusted odds ratio (OR) per calendar year and for each covariate. Sparse source and diagnosis categories were collapsed into an “Other” level to ensure stable estimation; antibiotics with rare or absent non-susceptibility were reported descriptively rather than modeled.

**Software and significance.** Analyses were performed in Python 3 (pandas, NumPy, SciPy, statsmodels). Statistical significance was defined as a two-sided p < 0.05. For the secondary antibiotic-specific MRSA-versus-MSSA comparisons, p-values were additionally adjusted for multiple comparisons using the Benjamini–Hochberg false discovery rate (FDR).

## 3. Results

### 3.1. Cohort derivation and isolate characteristics

The analytic cohort was large and the burden of methicillin resistance was substantial. A total of 4,194 *S. aureus* isolates from patients aged ≥12 years were available; no record involved a patient aged <12 years, and none lacked an organism classification or year of isolation, so no isolates required exclusion for missing essential variables (Table A in S1 File). These isolates originated from 3,122 unique patients, and in keeping with the analysis plan all qualifying isolates were retained without de-duplication. MRSA accounted for 2,231 isolates (53.2%) and MSSA for 1,963 (46.8%) (overall MRSA proportion 53.2%, 95% CI 51.7–54.7). Compared with MSSA, MRSA isolates were more frequently recovered from inpatients and proportionally more often from surgical wards, while the two groups were similar in age (median 48 vs 45 years; p = 0.17) and sex (Table 1). Standardized mean differences were small for all characteristics (all < 0.25), and the groups differed most clearly by era of isolation, consistent with a rising MRSA share over time.

**Table 1:** Baseline characteristics of included S. aureus isolates by MRSA/MSSA status.

Characteristic	MRSA (n = 2,231)	MSSA (n = 1,963)	p-value	SMD
No. of isolates	2231	1963		
Age, median (IQR), years	48 (31–68)	45 (31–67)	0.17	0.032
**Sex, n (%)**			0.20	0.040
Male	1599 (71.7)	1371 (69.8)		
Female	632 (28.3)	592 (30.2)		
**Setting, n (%)**			<0.001	0.133
INP	1975 (88.5)	1648 (84.0)		
OPD	256 (11.5)	315 (16.0)		
**Ward category, n (%)**			<0.001	0.144
ICU/ Intermediate ICU	497 (22.3)	436 (22.2)		
Medical Wards	698 (31.3)	596 (30.4)		
Surgical Wards	765 (34.3)	598 (30.5)		
Not Admitted/ Other	271 (12.1)	333 (17.0)		
**Age group (years), n (%)**			0.14	0.072
12-34	689 (30.9)	621 (31.6)		
35-49	468 (21.0)	452 (23.0)		
50-64	435 (19.5)	336 (17.1)		
>=65	639 (28.6)	554 (28.2)		
**Source category, n (%)**			0.002	0.159
Blood	304 (13.6)	243 (12.4)		
Respiratory	659 (29.5)	652 (33.2)		
Skin/ Soft Tissue/ Wound	860 (38.5)	671 (34.2)		
Urine/ Genitourinary	190 (8.5)	199 (10.1)		
Sterile Body Fluids	50 (2.2)	49 (2.5)		
Bone/ Tissue Biopsy	50 (2.2)	26 (1.3)		
Surveillance/ Screening Swab	53 (2.4)	38 (1.9)		
Line/ Catheter Tip	11 (0.5)	15 (0.8)		
Eye/ Ear/ ENT	14 (0.6)	21 (1.1)		
Unspecified/ Other	40 (1.8)	49 (2.5)		
**Diagnosis category, n (%)**			<0.001	0.200
Skin & Soft Tissue Infection	165 (7.4)	172 (8.8)		
Trauma/ Injury	196 (8.8)	217 (11.1)		
Respiratory	93 (4.2)	60 (3.1)		
Sepsis/ Bloodstream Infection	59 (2.6)	51 (2.6)		
Renal/ Genitourinary	104 (4.7)	88 (4.5)		
Neurological	67 (3.0)	70 (3.6)		
Malignancy	45 (2.0)	47 (2.4)		
Burns	33 (1.5)	40 (2.0)		
Bone & Joint/ Prosthetic Infection	32 (1.4)	25 (1.3)		
Cardiovascular	22 (1.0)	28 (1.4)		
Surgical/ Post-procedure	26 (1.2)	17 (0.9)		
Endocrine/ Metabolic	19 (0.9)	14 (0.7)		
Hematologic	9 (0.4)	11 (0.6)		
Gastrointestinal/ Hepatic	13 (0.6)	4 (0.2)		
Autoimmune/ Rheumatologic	3 (0.1)	11 (0.6)		
Other/ Miscellaneous	134 (6.0)	159 (8.1)		
Uninformative/ Missing	1211 (54.3)	949 (48.3)		
**Era, n (%)**			<0.001	0.229
2013-2016	493 (22.1)	621 (31.6)		
2017-2020	846 (37.9)	711 (36.2)		
2021-2024	892 (40.0)	631 (32.1)		

**Note:** Data are n (%) unless otherwise indicated. MRSA, methicillin-resistant Staphylococcus aureus; MSSA, methicillin-susceptible Staphylococcus aureus; IQR, interquartile range; INP, inpatient; OPD, outpatient; SMD, standardized mean difference. All qualifying isolates were analyzed without de-duplication (denominators: MRSA n = 2,231; MSSA n = 1,963); percentages are column percentages within each group. Patients were aged ≥12 years; the dataset contained no patients aged <12 years, so no pediatric/neonatal records were removed. p-values are from the Mann–Whitney U test (age) and the chi-square or Fisher exact test (categorical variables) and treat the isolate as the unit of analysis. Ward, source, and diagnosis use the pre-defined Ward Category, Source Category, and Diagnosis Category variables, not raw free-text fields.

### 3.2. Annual distribution of MRSA and MSSA

**Fig 1:**
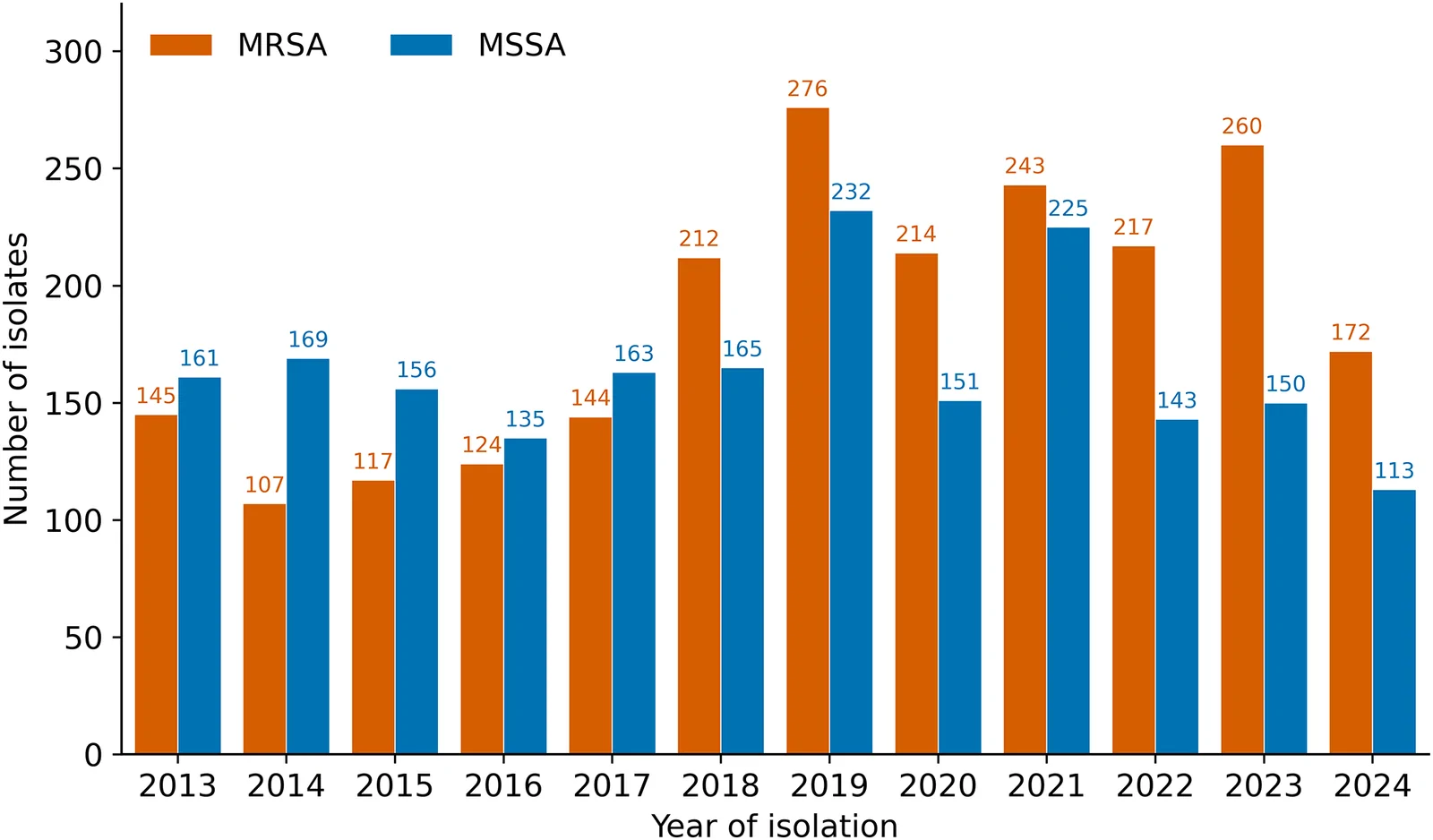
Annual distribution of MRSA and MSSA isolates, 2013–2024. Grouped bars show the number of methicillin-resistant (MRSA) and methicillin-susceptible (MSSA) Staphylococcus aureus isolates per year (all isolates; patients aged ≥12 years). The x-axis indicates the year of isolation and the y-axis the number of isolates.

Both the volume of *S. aureus* isolates and the predominance of MRSA increased over the study period. Annual isolate counts rose from 2013 and peaked in 2019, with MRSA increasingly outnumbering MSSA in later years (Fig 1; Table 2). The MRSA proportion was 47.4% in 2013, dipped to its lowest value of 38.8% in 2014, and then rose progressively to 63.4% in 2023, remaining elevated at 60.4% in 2024; the independent association between later calendar year and MRSA is quantified in the multivariable model (§3.7). Annual totals summed exactly to the analytic cohort of 4,194 isolates.

**Table 2:** Annual distribution of MRSA and MSSA, 2013–2024.

Year	Total S. aureus	MRSA, n	MSSA, n	MRSA, %	95% CI
2013	306	145	161	47.4	41.9–53.0
2014	276	107	169	38.8	33.2–44.6
2015	273	117	156	42.9	37.1–48.8
2016	259	124	135	47.9	41.9–53.9
2017	307	144	163	46.9	41.4–52.5
2018	377	212	165	56.2	51.2–61.2
2019	508	276	232	54.3	50.0–58.6
2020	365	214	151	58.6	53.5–63.6
2021	468	243	225	51.9	47.4–56.4
2022	360	217	143	60.3	55.1–65.2
2023	410	260	150	63.4	58.6–67.9
2024	285	172	113	60.4	54.6–65.9
**Total**	**4194**	**2231**	**1963**	**53.2**	**51.7–54.7**

Note: Annual denominator is the total number of S. aureus isolates (all isolates) for each year. MRSA percentage = MRSA/ total S. aureus for that year. 95% CI is the Wilson score binomial confidence interval for the MRSA proportion. Patients aged ≥12 years. MRSA, methicillin-resistant S. aureus; MSSA, methicillin-susceptible S. aureus; CI, confidence interval.

### 3.3. Temporal trends in antimicrobial non-susceptibility

**Fig 2:**
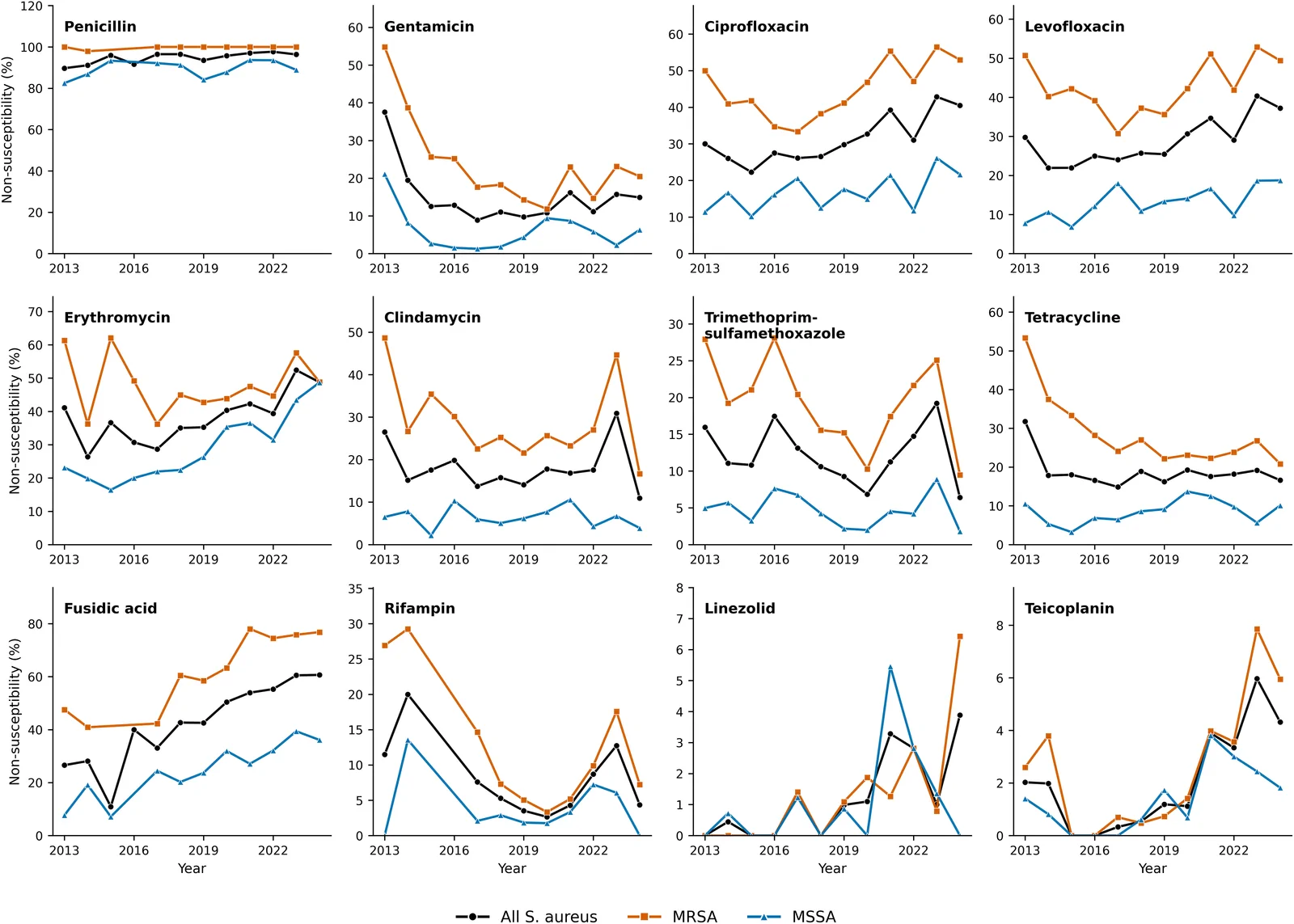
Annual antimicrobial non-susceptibility over time, by organism group, 2013–2024. Each panel shows the annual percentage of non-susceptible (intermediate or resistant) isolates among those tested for one antibiotic, plotted separately for all S. aureus, MRSA, and MSSA. Annual points are displayed for years with at least 20 tested isolates in a group to avoid unstable estimates from small denominators; trends were quantified by segmented log-linear (joinpoint) regression (Table 3). Vancomycin and daptomycin are not shown because no non-susceptibility was detected in either group.

Non-susceptibility trends diverged by agent, and several increases were driven as much by the methicillin-susceptible population as by MRSA. Over 2013–2024, non-susceptibility to fusidic acid rose significantly in all *S. aureus* and within both MRSA and MSSA (APC approximately +6 to +9% per year; all p ≤ 0.001), the most consistent increasing trend observed (Table 3; Fig 2). Non-susceptibility to the fluoroquinolones (ciprofloxacin and levofloxacin) and to erythromycin also increased, with the steepest rises among MSSA (for example, erythromycin in MSSA, APC + 9.1% per year, p < 0.001), indicating accrual of resistance traits in the methicillin-susceptible population. In contrast, gentamicin and tetracycline non-susceptibility declined, most clearly within MRSA (gentamicin MRSA APC −8.4% per year, p = 0.007; tetracycline MRSA APC −6.3% per year, p = 0.002). Penicillin non-susceptibility remained near-universal in MRSA and high in MSSA, with only minimal change over time (APC < 1% per year). Low-level non-susceptibility to linezolid and teicoplanin increased from a very low base (e.g., linezolid, all *S. aureus*, APC + 21.8% per year, p = 0.01); because confirmatory testing was not performed, these emerging signals should be interpreted cautiously. No vancomycin or daptomycin non-susceptibility was detected, so trends for these agents were not estimable (Table 3).

**Table 3:** Temporal trends (joinpoint/segmented log-linear) in antimicrobial non-susceptibility among S. aureus, overall and by methicillin-resistance status, 2013–2024.

Antibiotic	All S. aureus APC %/yr (95% CI); p	MRSA APC %/yr (95% CI); p	MSSA APC %/yr (95% CI); p
Penicillin	+0.53 (0.2 to 0.87) p = 0.013	+0.07 (–0.0 to 0.14) p = 0.091	+0.6 (–0.17 to 1.36) p = 0.164
Gentamicin	–6.61 (–12.06 to –0.82) ‡ p = 0.050	–8.4 (–12.98 to –3.58) ‡ p = 0.007	–8.17 (–16.94 to 1.52) ‡ p = 0.127
Ciprofloxacin	+4.9 (2.8 to 7.03) p < 0.001	+2.75 (0.49 to 5.05) ‡ p = 0.038	+4.73 (0.86 to 8.75) p = 0.037
Levofloxacin	+5.49 (3.19 to 7.83) p < 0.001	+2.59 (–0.04 to 5.3) ‡ p = 0.083	+5.45 (1.05 to 10.03) p = 0.035
Erythromycin	+4.08 (1.58 to 6.65) ‡ p = 0.009	–0.33 (–3.03 to 2.44) p = 0.816	+9.09 (6.48 to 11.76) ‡ p < 0.001
Clindamycin	+1.23 (–4.01 to 6.75) p = 0.662	–1.14 (–6.28 to 4.29) ‡ p = 0.684	–0.41 (–6.36 to 5.92) p = 0.898
Trimethoprim-sulfamethoxazole	+0.35 (–5.09 to 6.11) p = 0.904	–2.28 (–7.11 to 2.8) p = 0.393	–0.24 (–7.57 to 7.66) p = 0.951
Tetracycline	–2.53 (–5.48 to 0.52) ‡ p = 0.134	–6.31 (–9.1 to –3.43) ‡ p = 0.002	+3.84 (–1.91 to 9.93) p = 0.223
Fusidic acid	+8.14 (6.21 to 10.1) p < 0.001	+5.78 (3.39 to 8.23) p = 0.001	+9.32 (5.45 to 13.34) p < 0.001
Rifampin	–7.35 (–16.75 to 3.12) p = 0.200	–9.19 (–18.1 to 0.69) ‡ p = 0.105	–8.36 (–20.5 to 5.63) p = 0.263
Linezolid	+21.84 (7.89 to 37.6) p = 0.010	+27.02 (13.12 to 42.63) p = 0.002	+20.76 (0.4 to 45.25) p = 0.073
Teicoplanin	+16.3 (4.6 to 29.31) ‡ p = 0.019	+14.3 (2.01 to 28.07) ‡ p = 0.044	+12.76 (1.33 to 25.48) p = 0.052
Vancomycin	NE	NE	NE
Daptomycin	NE	NE	NE

**Note:** APC, annual percent change (the average yearly relative change in the non-susceptibility proportion); CI, confidence interval. Each estimate is from a segmented log-linear regression of the annual non-susceptibility proportion (non-susceptible/ tested, by year), weighted by the inverse variance of the log-proportion, with a Haldane continuity correction for zero-non-susceptibility years; non-susceptibility = intermediate or resistant. Denominators exclude not-reported/not-tested results, and annual points were restricted to years with ≥20 tested isolates in a group. A maximum of one joinpoint was permitted, tested by grid search with an F-test; ‡ marks series in which a statistically significant joinpoint was identified (gentamicin, ciprofloxacin, levofloxacin, erythromycin, clindamycin, tetracycline, rifampin, and teicoplanin in one or more groups). In each such series an unstable early-period change (small early-year denominators) preceded the later trend, so the whole-period APC is reported as the primary summary and segment-specific estimates are not emphasized. NE, not estimable (no non-susceptibility observed [vancomycin, daptomycin] or data too sparse for a stable model). Formal NCI Joinpoint software was not used; a reproducible segmented-regression implementation was applied. All isolates; patients aged ≥12 years. Era-stratified estimates and p-for-trend are provided in Table E in S1 File.

### 3.4. Distribution by setting, ward category, and source category

Methicillin resistance was concentrated in inpatient and higher-acuity settings. MRSA accounted for a larger share of *S. aureus* among inpatients than outpatients and was proportionally more common on surgical wards than on non-admitted services (Table 1). Setting-stratified annual distributions confirmed that the inpatient setting carried the majority of isolates and the higher MRSA proportion throughout the period, with only INP and OPD settings present in the data (Table B in S1 File). By specimen origin, MRSA proportions varied modestly across source categories, being somewhat lower among respiratory isolates and broadly similar across skin/soft-tissue, blood, and urinary sources over time (Table C in S1 File).

### 3.5. Antimicrobial non-susceptibility profiles of MRSA and MSSA

**Fig 3:**
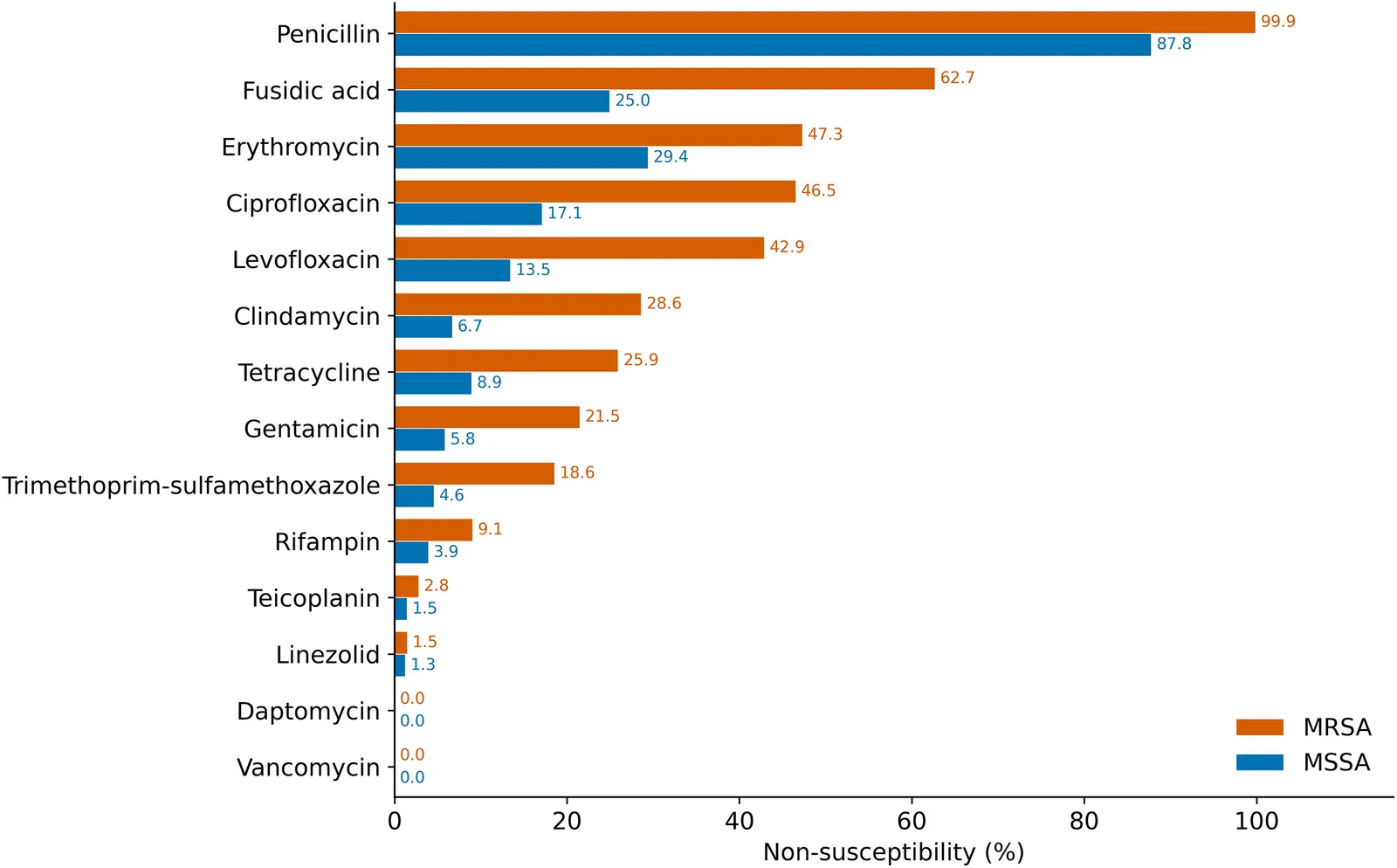
Antimicrobial non-susceptibility of MRSA versus MSSA for clinically relevant agents. Horizontal bars show the percentage of non-susceptible (intermediate or resistant) isolates among those tested, by organism group, for clinically relevant antibiotics (all isolates). Denominators differ by antibiotic because not-tested and not-reported results were excluded. Vancomycin and daptomycin showed no non-susceptibility in either group.

MRSA isolates were substantially more non-susceptible than MSSA across nearly every drug class, yet glycopeptides and oxazolidinones retained near-complete activity. Non-susceptibility was significantly higher in MRSA than MSSA for penicillin, the fluoroquinolones (ciprofloxacin and levofloxacin), erythromycin, clindamycin, gentamicin, trimethoprim–sulfamethoxazole, tetracycline, fusidic acid, and rifampin, with all comparisons remaining significant after FDR adjustment (Table 4; Fig 3). The largest absolute gaps were for fusidic acid and the fluoroquinolones (each ≈29–38 percentage points higher in MRSA). In contrast, no vancomycin or daptomycin non-susceptibility was detected in either group, and linezolid non-susceptibility was low and not significantly different between groups (1.5% vs 1.3%; p = 0.66); the latter should be interpreted cautiously in the absence of confirmatory testing. Full tested denominators and susceptible/non-susceptible/not-tested counts for every antibiotic are provided in Table D in S1 File.

**Table 4:** Antimicrobial non-susceptibility profile of MRSA versus MSSA.

Antibiotic	MRSA tested	MRSA NS, n (%)	MSSA tested	MSSA NS, n (%)	Δ%	p	q (FDR)
Penicillin	850	849 (99.9)	655	575 (87.8)	+12.1	<0.001	<0.001
Gentamicin	2113	454 (21.5)	1848	108 (5.8)	+15.6	<0.001	<0.001
Ciprofloxacin	1360	633 (46.5)	1291	221 (17.1)	+29.4	<0.001	<0.001
Levofloxacin	2093	898 (42.9)	1799	242 (13.5)	+29.5	<0.001	<0.001
Erythromycin	2028	960 (47.3)	1755	516 (29.4)	+17.9	<0.001	<0.001
Clindamycin	1890	541 (28.6)	1746	117 (6.7)	+21.9	<0.001	<0.001
Trimethoprim-sulfamethoxazole	2176	404 (18.6)	1896	87 (4.6)	+14.0	<0.001	<0.001
Tetracycline	1936	502 (25.9)	1621	145 (8.9)	+17.0	<0.001	<0.001
Fusidic acid	1514	949 (62.7)	1326	331 (25.0)	+37.7	<0.001	<0.001
Rifampin	1148	104 (9.1)	1017	40 (3.9)	+5.1	<0.001	<0.001
Linezolid	2106	31 (1.5)	1827	23 (1.3)	+0.2	0.663	0.663
Teicoplanin	2003	56 (2.8)	1713	25 (1.5)	+1.3	0.00762	0.00832
Vancomycin	2215	0 (0.0)	1946	0 (0.0)	0.0	NE	NE
Daptomycin	1164	0 (0.0)	1110	0 (0.0)	0.0	NE	NE

**Note:** NS, non-susceptible (= intermediate or resistant; in this dataset intermediate and resistant were recorded combined and could not be separated). Tested = number of isolates with a susceptible or non-susceptible result; not-reported, not-tested, missing, and blank values were excluded from the denominator and never counted as resistant. Δ% = absolute difference in non-susceptibility (MRSA minus MSSA). p-values are from the chi-square test, or the Fisher exact test when any expected cell count was < 5, and treat the isolate as the unit of analysis; q is the Benjamini–Hochberg FDR-adjusted p-value. NE, not estimable. Vancomycin and daptomycin showed no non-susceptibility in either group and are reported descriptively. Linezolid results should be interpreted cautiously because confirmatory testing was not performed. Oxacillin defines MRSA/MSSA status and is not shown. All isolates; patients aged ≥12 years.

### 3.6. Era-based antimicrobial non-susceptibility trends

Era-stratified analysis corroborated the annual joinpoint trends described in §3.3 and added p-for-trend estimates across three periods (2013–2016, 2017–2020, 2021–2024). Consistent with the annual models, MRSA fusidic-acid non-susceptibility rose from 44.3% to 76.6% (p for trend <0.001) and fluoroquinolone non-susceptibility increased across eras, whereas several other agents showed more modest or declining changes (Table E in S1 File). The low but rising non-susceptibility to linezolid (0.0% to 2.5% in MRSA) and teicoplanin (1.4% to 5.4% in MRSA) seen in the most recent era reinforces the emerging signals noted above and should be monitored prospectively rather than treated as established resistance.

### 3.7. Multivariable predictors of MRSA

Calendar year was the dominant independent predictor of methicillin resistance. In the multivariable GEE model (all isolates, clustered by MRN), each later calendar year was associated with higher odds of MRSA (adjusted OR 1.09, 95% CI 1.06–1.12), independent of patient and specimen characteristics (Table 5); this confirms the rising MRSA share noted descriptively in §3.2. A respiratory source was associated with lower odds of MRSA relative to skin/soft-tissue/wound specimens (adjusted OR 0.81), whereas a respiratory diagnosis was associated with higher odds (adjusted OR 1.44); age group, sex, setting, and ward category were not independently associated with MRSA after adjustment.

**Table 5:** Multivariable predictors of MRSA (all isolates; GEE logistic regression clustered by MRN).

Variable	Category (vs reference)	aOR	95% CI	p
**Calendar year (per year)**	continuous	1.09	1.06–1.12	<0.001
**Age group**	35-49 vs 12–34	1.01	0.83–1.22	0.94
	50-64 vs 12–34	1.15	0.93–1.42	0.189
	>=65 vs 12–34	1.11	0.93–1.34	0.256
**Sex**	Male vs Female	0.96	0.83–1.13	0.641
**Setting**	INP vs OPD	1.16	0.58–2.30	0.676
**Ward category**	ICU/Intermediate ICU vs Medical	1.03	0.84–1.25	0.796
	Surgical vs Medical	1.04	0.88–1.23	0.658
	Not Admitted/Other vs Medical	0.75	0.39–1.46	0.401
**Source category**	Blood vs Skin/Soft Tissue/Wound	0.91	0.74–1.11	0.355
	Respiratory vs Skin/Soft Tissue/Wound	0.81	0.69–0.96	0.0158
	Urine/GU vs Skin/Soft Tissue/Wound	0.83	0.64–1.07	0.148
	Other vs Skin/Soft Tissue/Wound	0.95	0.79–1.14	0.571
**Diagnosis category**	SSTI vs Uninformative/Missing	0.9	0.71–1.13	0.366
	Trauma/Injury vs Uninformative/Missing	0.92	0.75–1.14	0.473
	Respiratory vs Uninformative/Missing	1.44	1.05–1.99	0.0249
	Sepsis/BSI vs Uninformative/Missing	0.95	0.71–1.28	0.744
	Other vs Uninformative/Missing	0.94	0.82–1.08	0.401

**Note:** Outcome = MRSA versus MSSA. Estimates are adjusted odds ratios (aOR) with 95% CI from a multivariable GEE logistic regression on all isolates, clustered by MRN with an exchangeable working correlation (patients aged ≥12 years). Reference categories: age group 12–34 years; sex female; setting OPD; Ward Category Medical Wards; Source Category Skin/Soft Tissue/Wound; Diagnosis Category Uninformative/Missing. Sparse source and diagnosis categories were collapsed into “Other” for stable estimation. Ward, source, and diagnosis use the pre-defined Category variables, not raw free-text fields. De-duplicated sensitivity analyses gave concordant calendar-year effects (Table F in S1 File).

### 3.8. Sensitivity analyses

The principal findings were robust to the handling of repeated isolates. The overall MRSA proportion and the increasing calendar-year trend were materially unchanged across the primary all-isolate analysis, a first-isolate-per-patient-per-year de-duplicated analysis, and a first-isolate-per-patient (whole-period) analysis (Table F in S1 File). The per-year odds ratios were closely concordant (approximately 1.08–1.09 across analyses), indicating that retaining repeat isolates from the same patient did not materially alter the observed temporal findings.

## 4. Discussion

In this single-center, laboratory-based surveillance of 4,194 *S. aureus* isolates from the Aseer region over 2013–2024, MRSA shifted from a minority of isolates in the earliest years (47.4% in 2013, falling to 38.8% in 2014) to a stable majority in the later period, accounting for 53.2% of isolates overall and rising to 60.4% by 2024, with each later calendar year independently associated with MRSA after adjustment (adjusted odds ratio 1.09 per year). This rising local MRSA proportion is consistent with regional Saudi reports of a substantial and increasing MRSA burden [6,12] and lies within the wide range described nationally [11]. MRSA was significantly more non-susceptible than MSSA to penicillin, the fluoroquinolones, erythromycin, clindamycin, gentamicin, trimethoprim–sulfamethoxazole, tetracycline, fusidic acid, and rifampin, with all differences persisting after false-discovery-rate adjustment, underscoring the broader multidrug phenotype that accompanies methicillin resistance [9].

Several non-susceptibility trends were noteworthy. Non-susceptibility to fusidic acid increased in both MRSA and MSSA and was the most consistent rising trend, while fluoroquinolone and erythromycin non-susceptibility also increased, with the steepest erythromycin rise occurring in MSSA, indicating that the accrual of resistance was not confined to MRSA. In contrast, gentamicin and tetracycline non-susceptibility declined, most clearly within MRSA. Rifampin non-susceptibility, although higher in MRSA than MSSA cross-sectionally, also showed a declining temporal trend in MRSA that did not reach statistical significance. Because these are surveillance data without linked antimicrobial-consumption information, the drivers of these trends are interpreted cautiously; although outpatient antibiotic use is broadly associated with community resistance [16], our dataset cannot establish such a link and we therefore avoid causal or community-versus-hospital attributions.

For susceptible isolates, agents such as trimethoprim–sulfamethoxazole, tetracyclines, and clindamycin may remain useful oral options for skin and soft-tissue infection; however, the higher non-susceptibility observed in MRSA (for example trimethoprim–sulfamethoxazole [18.6% versus 4.6%] and clindamycin [28.6% versus 6.7%]) means empirical choices should be guided by local susceptibility data and confirmed by testing, and were higher than in a recent United States outpatient series [17]. Empirical management of invasive infection should continue to follow susceptibility-guided therapy and local guidelines rather than surveillance data alone, which lack information on syndrome, severity, and source control.

No vancomycin or daptomycin non-susceptibility was detected in either group, which is reassuring and consistent with these agents retaining activity against *S. aureus* in many settings [18]. Low-level and increasing non-susceptibility to linezolid and teicoplanin was observed from a very low base, and teicoplanin non-susceptibility was significantly higher in MRSA than MSSA (2.8% versus 1.5%); because confirmatory testing was not performed and minimum inhibitory concentration (MIC) distributions were not available in this dataset, these signals should be regarded as hypothesis-generating and monitored prospectively rather than treated as established resistance [19,20].

MRSA was concentrated in inpatients, yet the multivariable model found no independent association of patient-care setting, ward category, age, or sex with MRSA after adjustment for calendar year and specimen characteristics. The broadly similar MRSA non-susceptibility across settings is compatible with overlap between hospital- and community-circulating strains, but this laboratory dataset cannot distinguish HA-MRSA from CA-MRSA, and molecular typing would be required to test that hypothesis; integrated infection prevention and unified surveillance remain appropriate regardless [21].

Future work should incorporate molecular and genomic characterization to define clonal lineages and resistance determinants, link microbiological data to clinical outcomes and antimicrobial consumption, and extend surveillance across multiple Saudi centers to assess generalizability and the impact of stewardship interventions.

### 4.1. Limitations

This study has several limitations. It was conducted at a single tertiary-care center in the Aseer region using routinely collected, laboratory-based data; the findings may not generalize to other regions of Saudi Arabia or to community settings, and they could not be linked to clinical outcomes such as treatment failure, recurrence, or mortality, or to antimicrobial-consumption data. Susceptibility was available only as a combined non-susceptible (intermediate-or-resistant) category, and MIC distributions were not available, so MIC-based trends for vancomycin, daptomycin, or other agents could not be examined. Confirmatory testing for linezolid was not performed, so the low-level linezolid non-susceptibility signal is unconfirmed. Molecular confirmation was performed on only 100 phenotypically defined MRSA isolates, all drawn from inpatients in the final two surveillance years, using an assay that confirms MRSA but does not perform SCCmec typing or whole-genome sequencing; this subset served solely as a confirmatory quality-assurance check and is not representative of the full 2013–2024 study period or of outpatient isolates, precluding inferences about clonal structure, resistance mechanisms, or the molecular epidemiology of the wider collection. Susceptibility testing spanned two automated platforms and successive CLSI breakpoint revisions over the study period, which may introduce some measurement variability. Finally, repeated isolates from the same patient were retained in the primary analysis; although within-patient correlation was addressed using generalized estimating equations and de-duplicated sensitivity analyses gave concordant results, the isolate-level design is not fully patient-level.

### 4.2. Conclusion

This decade-long surveillance documents a clear epidemiological shift in the Aseer region: MRSA now constitutes the majority of clinical S. aureus and carries substantially higher multidrug non-susceptibility than MSSA, even as vancomycin and daptomycin retain complete in vitro activity. Rising fusidic-acid and fluoroquinolone non-susceptibility in both MRSA and MSSA indicates that resistance pressure extends beyond methicillin resistance alone, while an emerging, unconfirmed linezolid signal warrants vigilance. These locally specific, longitudinal data establish an evidence base to guide empirical therapy and to reinforce, rather than replace, antimicrobial stewardship. Sustained surveillance, confirmatory testing of last-resort-agent signals, and multicenter validation are now needed to preserve the agents that remain effective.

## Supporting information

S1 File Supplementary tables. Table A: Data cleaning and cohort derivation. Table B: Annual MRSA/MSSA distribution by setting (INP vs OPD). Table C: Annual MRSA/MSSA distribution by Source Category. Table D: Full antimicrobial susceptibility denominators by antibiotic and organism group. Table E: Era-based antimicrobial non-susceptibility trends (clinically relevant agents). Table F: Sensitivity analysis: primary all-isolate analysis versus de-duplicated cohorts.
(DOCX)
