# Supplementary material for: Methicillin resistance and antimicrobial non-susceptibility trends among Staphylococcus aureus clinical isolates in Aseer, Saudi Arabia (2013–2024): A retrospective laboratory-based surveillance study
Source: PLoS One. 2026 Sep 15;21(9):e0357789. doi: 10.1371/journal.pone.0357789 (PMC13577330; doi:10.1371/journal.pone.0357789)
Supplement: Supplementary file 1 [file pone.0357789.s001.docx]

**S1 File. Supplementary tables.**

**Table A.** Data cleaning and cohort derivation

| Step | N |
| --- | --- |
| Raw dataset records | 4194 |
| Records aged <12 years (excluded) | 0 |
| Records aged >=12 years | 4194 |
| Excluded: missing/!=MRSA-MSSA organism status | 0 |
| Excluded: missing year of isolation | 0 |
| PRIMARY analytic cohort: ALL isolates (no de-duplication) | 4194 |
| of which MRSA | 2231 |
| of which MSSA | 1963 |
| Unique patients (MRN) represented | 3122 |
| Repeat isolates retained (patients with >1 isolate over period) | 1072 |
| Sensitivity cohort: first isolate per patient per calendar year | 3272 |
| Sensitivity cohort: first isolate per patient (whole period) | 3122 |
| *Footnote. Derivation of the analytic cohort. The dataset contained no patients aged <12 years; the age ≥12 years criterion therefore removed no records and is not a pediatric/neonatal exclusion. No isolates were excluded for missing organism (MRSA/MSSA) status or missing year. The primary analytic cohort comprised all qualifying isolates with no de-duplication; the first-isolate-per-patient-per-calendar-year and first-isolate-per-patient (whole-period) cohorts are pre-specified de-duplicated sensitivity populations. Within-patient correlation was addressed in the regression models by GEE clustered by MRN. MRN, medical record number; GEE, generalized estimating equations.* | |

**Table B.** Annual MRSA/MSSA distribution by setting (INP vs OPD)

| Year | Setting | Total S. aureus | MRSA, n | MSSA, n | MRSA, % | 95% CI |
| --- | --- | --- | --- | --- | --- | --- |
| 2013 | INP | 291 | 139 | 152 | 47.8 | 42.1-53.5 |
| 2013 | OPD | 15 | 6 | 9 | 40.0 | 19.8-64.3 |
| 2014 | INP | 255 | 101 | 154 | 39.6 | 33.8-45.7 |
| 2014 | OPD | 21 | 6 | 15 | 28.6 | 13.8-50.0 |
| 2015 | INP | 241 | 110 | 131 | 45.6 | 39.5-52.0 |
| 2015 | OPD | 32 | 7 | 25 | 21.9 | 11.0-38.8 |
| 2016 | INP | 244 | 122 | 122 | 50.0 | 43.8-56.2 |
| 2016 | OPD | 15 | 2 | 13 | 13.3 | 3.7-37.9 |
| 2017 | INP | 264 | 129 | 135 | 48.9 | 42.9-54.9 |
| 2017 | OPD | 43 | 15 | 28 | 34.9 | 22.4-49.8 |
| 2018 | INP | 338 | 192 | 146 | 56.8 | 51.5-62.0 |
| 2018 | OPD | 39 | 20 | 19 | 51.3 | 36.2-66.1 |
| 2019 | INP | 442 | 240 | 202 | 54.3 | 49.6-58.9 |
| 2019 | OPD | 66 | 36 | 30 | 54.5 | 42.6-66.0 |
| 2020 | INP | 300 | 182 | 118 | 60.7 | 55.0-66.0 |
| 2020 | OPD | 65 | 32 | 33 | 49.2 | 37.5-61.1 |
| 2021 | INP | 391 | 217 | 174 | 55.5 | 50.5-60.3 |
| 2021 | OPD | 77 | 26 | 51 | 33.8 | 24.2-44.9 |
| 2022 | INP | 288 | 183 | 105 | 63.5 | 57.8-68.9 |
| 2022 | OPD | 72 | 34 | 38 | 47.2 | 36.1-58.6 |
| 2023 | INP | 338 | 216 | 122 | 63.9 | 58.7-68.8 |
| 2023 | OPD | 72 | 44 | 28 | 61.1 | 49.6-71.5 |
| 2024 | INP | 231 | 144 | 87 | 62.3 | 55.9-68.3 |
| 2024 | OPD | 54 | 28 | 26 | 51.9 | 38.9-64.6 |
| *Footnote. All isolates (no de-duplication; patients aged ≥12 years). Setting contains only inpatient (INP) and outpatient (OPD); no emergency/ER/EMR category exists in the data. 95% CI = Wilson score binomial confidence interval for the MRSA proportion. NE, not estimable (no isolates in that stratum-year).* | | | | | | |

**Table C.** Annual MRSA/MSSA distribution by Source Category

| Year | Source Category | Total | MRSA, n | MSSA, n | MRSA, % | 95% CI |
| --- | --- | --- | --- | --- | --- | --- |
| 2013 | Blood | 40 | 12 | 28 | 30.0 | 18.1-45.4 |
| 2013 | Respiratory | 107 | 45 | 62 | 42.1 | 33.1-51.5 |
| 2013 | Skin / Soft Tissue / Wound | 110 | 68 | 42 | 61.8 | 52.5-70.4 |
| 2013 | Urine / Genitourinary | 13 | 3 | 10 | 23.1 | 8.2-50.3 |
| 2013 | Other sources | 36 | 17 | 19 | 47.2 | 32.0-63.0 |
| 2014 | Blood | 27 | 11 | 16 | 40.7 | 24.5-59.3 |
| 2014 | Respiratory | 81 | 23 | 58 | 28.4 | 19.7-39.0 |
| 2014 | Skin / Soft Tissue / Wound | 117 | 48 | 69 | 41.0 | 32.5-50.1 |
| 2014 | Urine / Genitourinary | 13 | 3 | 10 | 23.1 | 8.2-50.3 |
| 2014 | Other sources | 38 | 22 | 16 | 57.9 | 42.2-72.1 |
| 2015 | Blood | 30 | 18 | 12 | 60.0 | 42.3-75.4 |
| 2015 | Respiratory | 82 | 37 | 45 | 45.1 | 34.8-55.9 |
| 2015 | Skin / Soft Tissue / Wound | 107 | 44 | 63 | 41.1 | 32.3-50.6 |
| 2015 | Urine / Genitourinary | 4 | 1 | 3 | 25.0 | 4.6-69.9 |
| 2015 | Other sources | 50 | 17 | 33 | 34.0 | 22.4-47.8 |
| 2016 | Blood | 21 | 11 | 10 | 52.4 | 32.4-71.7 |
| 2016 | Respiratory | 105 | 49 | 56 | 46.7 | 37.4-56.2 |
| 2016 | Skin / Soft Tissue / Wound | 95 | 53 | 42 | 55.8 | 45.8-65.4 |
| 2016 | Urine / Genitourinary | 8 | 2 | 6 | 25.0 | 7.1-59.1 |
| 2016 | Other sources | 30 | 9 | 21 | 30.0 | 16.7-47.9 |
| 2017 | Blood | 36 | 14 | 22 | 38.9 | 24.8-55.1 |
| 2017 | Respiratory | 120 | 59 | 61 | 49.2 | 40.4-58.0 |
| 2017 | Skin / Soft Tissue / Wound | 111 | 54 | 57 | 48.6 | 39.6-57.8 |
| 2017 | Urine / Genitourinary | 15 | 6 | 9 | 40.0 | 19.8-64.3 |
| 2017 | Other sources | 25 | 11 | 14 | 44.0 | 26.7-62.9 |
| 2018 | Blood | 54 | 34 | 20 | 63.0 | 49.6-74.6 |
| 2018 | Respiratory | 121 | 64 | 57 | 52.9 | 44.0-61.6 |
| 2018 | Skin / Soft Tissue / Wound | 140 | 76 | 64 | 54.3 | 46.0-62.3 |
| 2018 | Urine / Genitourinary | 26 | 15 | 11 | 57.7 | 38.9-74.5 |
| 2018 | Other sources | 36 | 23 | 13 | 63.9 | 47.6-77.5 |
| 2019 | Blood | 68 | 40 | 28 | 58.8 | 47.0-69.7 |
| 2019 | Respiratory | 187 | 87 | 100 | 46.5 | 39.5-53.7 |
| 2019 | Skin / Soft Tissue / Wound | 157 | 93 | 64 | 59.2 | 51.4-66.6 |
| 2019 | Urine / Genitourinary | 60 | 32 | 28 | 53.3 | 40.9-65.4 |
| 2019 | Other sources | 36 | 24 | 12 | 66.7 | 50.3-79.8 |
| 2020 | Blood | 51 | 27 | 24 | 52.9 | 39.5-65.9 |
| 2020 | Respiratory | 103 | 67 | 36 | 65.0 | 55.4-73.6 |
| 2020 | Skin / Soft Tissue / Wound | 138 | 81 | 57 | 58.7 | 50.4-66.6 |
| 2020 | Urine / Genitourinary | 49 | 27 | 22 | 55.1 | 41.3-68.1 |
| 2020 | Other sources | 24 | 12 | 12 | 50.0 | 31.4-68.6 |
| 2021 | Blood | 60 | 35 | 25 | 58.3 | 45.7-69.9 |
| 2021 | Respiratory | 119 | 57 | 62 | 47.9 | 39.1-56.8 |
| 2021 | Skin / Soft Tissue / Wound | 171 | 97 | 74 | 56.7 | 49.2-63.9 |
| 2021 | Urine / Genitourinary | 69 | 29 | 40 | 42.0 | 31.1-53.8 |
| 2021 | Other sources | 49 | 25 | 24 | 51.0 | 37.5-64.4 |
| 2022 | Blood | 59 | 43 | 16 | 72.9 | 60.4-82.6 |
| 2022 | Respiratory | 112 | 69 | 43 | 61.6 | 52.4-70.1 |
| 2022 | Skin / Soft Tissue / Wound | 114 | 67 | 47 | 58.8 | 49.6-67.4 |
| 2022 | Urine / Genitourinary | 47 | 20 | 27 | 42.6 | 29.5-56.7 |
| 2022 | Other sources | 28 | 18 | 10 | 64.3 | 45.8-79.3 |
| 2023 | Blood | 52 | 32 | 20 | 61.5 | 48.0-73.5 |
| 2023 | Respiratory | 111 | 62 | 49 | 55.9 | 46.6-64.7 |
| 2023 | Skin / Soft Tissue / Wound | 154 | 102 | 52 | 66.2 | 58.5-73.2 |
| 2023 | Urine / Genitourinary | 59 | 40 | 19 | 67.8 | 55.1-78.3 |
| 2023 | Other sources | 34 | 24 | 10 | 70.6 | 53.8-83.2 |
| 2024 | Blood | 49 | 27 | 22 | 55.1 | 41.3-68.1 |
| 2024 | Respiratory | 63 | 40 | 23 | 63.5 | 51.1-74.3 |
| 2024 | Skin / Soft Tissue / Wound | 117 | 77 | 40 | 65.8 | 56.8-73.8 |
| 2024 | Urine / Genitourinary | 26 | 12 | 14 | 46.2 | 28.8-64.5 |
| 2024 | Other sources | 30 | 16 | 14 | 53.3 | 36.1-69.8 |
| *Footnote. All isolates (no de-duplication; patients aged ≥12 years). Analysis uses the pre-defined Source Category variable, not raw free-text source. Less frequent source categories (sterile body fluids, bone/tissue biopsy, surveillance/screening swab, line/catheter tip, eye/ear/ENT, unspecified/other) are combined as “Other sources” to keep stratum denominators interpretable. 95% CI = Wilson score interval. NE, not estimable.* | | | | | | |

**Table D.** Full antimicrobial susceptibility denominators by antibiotic and organism group

| Antibiotic | MRSA tested | MRSA S | MRSA NS | MRSA NT | MSSA tested | MSSA S | MSSA NS | MSSA NT |
| --- | --- | --- | --- | --- | --- | --- | --- | --- |
| amikacin | 17 | 1 | 16 | 2214 | 22 | 21 | 1 | 1941 |
| amoxicillin | 7 | 1 | 6 | 2224 | 7 | 5 | 2 | 1956 |
| amphotericin | 0 | 0 | 0 | 2231 | 1 | 1 | 0 | 1962 |
| ampicillin | 248 | 4 | 244 | 1983 | 310 | 64 | 246 | 1653 |
| amptam | 28 | 0 | 28 | 2203 | 33 | 25 | 8 | 1930 |
| augmentin | 391 | 13 | 378 | 1840 | 1287 | 1261 | 26 | 676 |
| azithromycin | 1173 | 619 | 554 | 1058 | 1068 | 759 | 309 | 895 |
| azlocillin | 0 | 0 | 0 | 2231 | 1 | 0 | 1 | 1962 |
| aztreonam | 2 | 0 | 2 | 2229 | 6 | 1 | 5 | 1957 |
| bactrim | 2176 | 1772 | 404 | 55 | 1896 | 1809 | 87 | 67 |
| cefotaxime | 34 | 3 | 31 | 2197 | 48 | 45 | 3 | 1915 |
| cefazolin | 3 | 1 | 2 | 2228 | 5 | 5 | 0 | 1958 |
| cefdinir | 1 | 0 | 1 | 2230 | 1 | 1 | 0 | 1962 |
| cefepime | 32 | 4 | 28 | 2199 | 42 | 39 | 3 | 1921 |
| cefoxitin | 74 | 0 | 74 | 2157 | 56 | 56 | 0 | 1907 |
| ceftazidime | 1 | 0 | 1 | 2230 | 1 | 0 | 1 | 1962 |
| ceftriaxone | 21 | 4 | 17 | 2210 | 26 | 24 | 2 | 1937 |
| cefuroxime | 19 | 0 | 19 | 2212 | 28 | 23 | 5 | 1935 |
| cephalothin | 41 | 17 | 24 | 2190 | 85 | 83 | 2 | 1878 |
| chloramphenicol | 63 | 55 | 8 | 2168 | 74 | 72 | 2 | 1889 |
| ciprofloxacin | 1360 | 727 | 633 | 871 | 1291 | 1070 | 221 | 672 |
| clarithromycin | 37 | 23 | 14 | 2194 | 37 | 28 | 9 | 1926 |
| clindamycin | 1890 | 1349 | 541 | 341 | 1746 | 1629 | 117 | 217 |
| colistin | 0 | 0 | 0 | 2231 | 6 | 6 | 0 | 1957 |
| daptomycin | 1164 | 1164 | 0 | 1067 | 1110 | 1110 | 0 | 853 |
| ertapenem | 0 | 0 | 0 | 2231 | 1 | 1 | 0 | 1962 |
| erythromycin | 2028 | 1068 | 960 | 203 | 1755 | 1239 | 516 | 208 |
| fosfomycin | 1246 | 1177 | 69 | 985 | 1131 | 1110 | 21 | 832 |
| fusidic_acid | 1514 | 565 | 949 | 717 | 1326 | 995 | 331 | 637 |
| gemifloxacin | 3 | 1 | 2 | 2228 | 1 | 1 | 0 | 1962 |
| gentamicin | 2113 | 1659 | 454 | 118 | 1848 | 1740 | 108 | 115 |
| imipenem | 201 | 12 | 189 | 2030 | 1065 | 1052 | 13 | 898 |
| inducible_clindamycin | 88 | 0 | 88 | 2143 | 19 | 1 | 18 | 1944 |
| kanamycin_synergy | 1 | 0 | 1 | 2230 | 0 | 0 | 0 | 1963 |
| levofloxacin | 2093 | 1195 | 898 | 138 | 1799 | 1557 | 242 | 164 |
| lincomycin | 1 | 1 | 0 | 2230 | 2 | 2 | 0 | 1961 |
| linezolid | 2106 | 2075 | 31 | 125 | 1827 | 1804 | 23 | 136 |
| meropenem | 6 | 1 | 5 | 2225 | 12 | 12 | 0 | 1951 |
| mezlocillin | 2 | 0 | 2 | 2229 | 5 | 2 | 3 | 1958 |
| moxifloxacin | 1879 | 1122 | 757 | 352 | 1530 | 1340 | 190 | 433 |
| mupirocin | 1049 | 987 | 62 | 1182 | 978 | 955 | 23 | 985 |
| nalidixic_acid | 1 | 0 | 1 | 2230 | 2 | 0 | 2 | 1961 |
| netilmicin | 35 | 32 | 3 | 2196 | 33 | 31 | 2 | 1930 |
| nitrofurantoin | 609 | 603 | 6 | 1622 | 421 | 420 | 1 | 1542 |
| norfloxacin | 4 | 1 | 3 | 2227 | 4 | 2 | 2 | 1959 |
| novobiocin | 0 | 0 | 0 | 2231 | 1 | 1 | 0 | 1962 |
| ofloxacin | 2 | 1 | 1 | 2229 | 1 | 1 | 0 | 1962 |
| oxacillin | 2231 | 0 | 2231 | 0 | 1963 | 1963 | 0 | 0 |
| oxacillin_methicillin | 0 | 0 | 0 | 2231 | 1 | 1 | 0 | 1962 |
| pefloxacin | 2 | 0 | 2 | 2229 | 3 | 1 | 2 | 1960 |
| penicillin | 850 | 1 | 849 | 1381 | 655 | 80 | 575 | 1308 |
| piperacillin | 7 | 0 | 7 | 2224 | 7 | 5 | 2 | 1956 |
| tazocin | 5 | 1 | 4 | 2226 | 10 | 10 | 0 | 1953 |
| rifampin | 1148 | 1044 | 104 | 1083 | 1017 | 977 | 40 | 946 |
| streptomycin | 1 | 0 | 1 | 2230 | 0 | 0 | 0 | 1963 |
| synercid | 1074 | 991 | 83 | 1157 | 930 | 906 | 24 | 1033 |
| teicoplanin | 2003 | 1947 | 56 | 228 | 1713 | 1688 | 25 | 250 |
| tetracycline | 1936 | 1434 | 502 | 295 | 1621 | 1476 | 145 | 342 |
| ticarcillin | 1 | 0 | 1 | 2230 | 4 | 4 | 0 | 1959 |
| ticar_clav | 2 | 2 | 0 | 2229 | 1 | 0 | 1 | 1962 |
| tigecycline | 710 | 709 | 1 | 1521 | 496 | 495 | 1 | 1467 |
| tobramycin | 630 | 524 | 106 | 1601 | 427 | 374 | 53 | 1536 |
| vancomycin | 2215 | 2215 | 0 | 16 | 1946 | 1946 | 0 | 17 |
| voriconazole | 1 | 1 | 0 | 2230 | 0 | 0 | 0 | 1963 |
| *Footnote. S, susceptible; NS, non-susceptible; NT, not tested / not reported (excluded from the tested denominator). Tested = S + NS. Intermediate (I) and resistant (R) results were recorded combined as a single non-susceptible (NS) code in the source laboratory export and could not be separated; therefore separate I and R columns are not estimable and NS represents intermediate-or-resistant. Antibiotics never tested for these isolates (all results not reported) are omitted; agents are shown where ≥1 isolate had a susceptible or non-susceptible result. Counts are from all isolates (no de-duplication; patients aged ≥12 years). Oxacillin defines MRSA/MSSA status and is not shown as an outcome.* | | | | | | | | |

**Table E.** Era-based antimicrobial non-susceptibility trends (clinically relevant agents)

| Antibiotic | Group | 2013–2016, n/N (%) | 2017–2020, n/N (%) | 2021–2024, n/N (%) | p-trend |
| --- | --- | --- | --- | --- | --- |
| Penicillin | MRSA | 134/135 (99.3%) | 361/361 (100.0%) | 354/354 (100.0%) | 0.0777 |
| Penicillin | MSSA | 170/198 (85.9%) | 215/243 (88.5%) | 190/214 (88.8%) | 0.37 |
| Gentamicin | MRSA | 152/429 (35.4%) | 126/828 (15.2%) | 176/856 (20.6%) | <0.001 |
| Gentamicin | MSSA | 42/548 (7.7%) | 29/700 (4.1%) | 37/600 (6.2%) | 0.311 |
| Ciprofloxacin | MRSA | 120/281 (42.7%) | 207/506 (40.9%) | 306/573 (53.4%) | <0.001 |
| Ciprofloxacin | MSSA | 48/359 (13.4%) | 78/471 (16.6%) | 95/461 (20.6%) | 0.00592 |
| Levofloxacin | MRSA | 162/383 (42.3%) | 311/843 (36.9%) | 425/867 (49.0%) | <0.001 |
| Levofloxacin | MSSA | 46/483 (9.5%) | 99/707 (14.0%) | 97/609 (15.9%) | 0.00243 |
| Erythromycin | MRSA | 168/318 (52.8%) | 355/835 (42.5%) | 437/875 (49.9%) | 0.741 |
| Erythromycin | MSSA | 88/433 (20.3%) | 185/702 (26.4%) | 243/620 (39.2%) | <0.001 |
| Clindamycin | MRSA | 141/396 (35.6%) | 174/735 (23.7%) | 226/759 (29.8%) | 0.204 |
| Clindamycin | MSSA | 34/482 (7.1%) | 42/679 (6.2%) | 41/585 (7.0%) | 0.99 |
| Trimethoprim-sulfamethoxazole | MRSA | 109/450 (24.2%) | 126/844 (14.9%) | 169/882 (19.2%) | 0.141 |
| Trimethoprim-sulfamethoxazole | MSSA | 30/564 (5.3%) | 26/710 (3.7%) | 31/622 (5.0%) | 0.818 |
| Tetracycline | MRSA | 111/292 (38.0%) | 200/835 (24.0%) | 191/809 (23.6%) | <0.001 |
| Tetracycline | MSSA | 23/353 (6.5%) | 65/694 (9.4%) | 57/574 (9.9%) | 0.0959 |
| Fusidic acid | MRSA | 78/176 (44.3%) | 468/812 (57.6%) | 403/526 (76.6%) | <0.001 |
| Fusidic acid | MSSA | 31/242 (12.8%) | 167/674 (24.8%) | 133/410 (32.4%) | <0.001 |
| Rifampin | MRSA | 22/71 (31.0%) | 34/526 (6.5%) | 48/551 (8.7%) | 0.003 |
| Rifampin | MSSA | 14/102 (13.7%) | 10/477 (2.1%) | 16/438 (3.7%) | 0.00534 |
| Linezolid | MRSA | 0/391 (0.0%) | 9/841 (1.1%) | 22/874 (2.5%) | <0.001 |
| Linezolid | MSSA | 1/497 (0.2%) | 4/709 (0.6%) | 18/621 (2.9%) | <0.001 |
| Teicoplanin | MRSA | 5/346 (1.4%) | 7/838 (0.8%) | 44/819 (5.4%) | <0.001 |
| Teicoplanin | MSSA | 2/434 (0.5%) | 6/703 (0.9%) | 17/576 (3.0%) | <0.001 |
| Vancomycin | MRSA | 0/480 (0.0%) | 0/845 (0.0%) | 0/890 (0.0%) | NE |
| Vancomycin | MSSA | 0/605 (0.0%) | 0/710 (0.0%) | 0/631 (0.0%) | NE |
| Daptomycin | MRSA | 0/190 (0.0%) | 0/479 (0.0%) | 0/495 (0.0%) | NE |
| Daptomycin | MSSA | 0/246 (0.0%) | 0/455 (0.0%) | 0/409 (0.0%) | NE |
| *Footnote. Values are non-susceptible / tested (%) per era. p-trend is the Cochran–Armitage test for linear trend across the three ordered eras. Tested denominators exclude not-reported/not-tested results; non-susceptibility = intermediate or resistant. NE, not estimable (no non-susceptibility observed, e.g., vancomycin and daptomycin, which are reported descriptively). Low-level linezolid and teicoplanin non-susceptibility should be interpreted cautiously because confirmatory testing was not performed. All isolates (no de-duplication), patients aged ≥12 years.* | | | | | |

**Table F.** Sensitivity analysis: primary all-isolate analysis versus de-duplicated cohorts

| Outcome | All isolates (primary) | First isolate/patient/year | First-ever/patient | Interpretation |
| --- | --- | --- | --- | --- |
| Overall MRSA proportion | 53.2% (51.7-54.7); n=4194 | 51.9% (50.2-53.6); n=3272 | 51.7% (49.9-53.4); n=3122 | Consistent across de-duplication strategies |
| MRSA trend, OR per calendar year (unadjusted) | 1.086 (1.061-1.111) [GEE] | 1.081 (1.058-1.104) | 1.084 (1.060-1.108) | Direction & significance unchanged |
| MRSA trend, adjusted OR per year | 1.091 (1.065-1.118) [GEE] | - | - | Conclusions unchanged after adjustment |
| *Footnote. Comparison of the overall MRSA proportion and the calendar-year trend across analytic strategies. Primary = all isolates with no de-duplication (trend odds ratios from GEE clustered by MRN, exchangeable correlation); first isolate/patient/year = one isolate per patient per calendar year; first-ever/patient = first isolate per patient across the whole study period (these two use ordinary logistic regression). Odds ratios are per calendar year with 95% CI. Concordance across analyses indicates the temporal trend is not an artifact of retaining repeat isolates. GEE, generalized estimating equations; CI, confidence interval.* | | | | |
