# Supplementary material for: Methicillin resistance and antimicrobial non-susceptibility trends among Staphylococcus aureus clinical isolates in Aseer, Saudi Arabia (2013–2024): A retrospective laboratory-based surveillance study
Source: PLoS One. 2026 Sep 15;21(9):e0357789. doi: 10.1371/journal.pone.0357789 (PMC13577330; doi:10.1371/journal.pone.0357789)
Supplement: Supplementary file 2 [file pone.0357789.s002.docx]

**Response to Reviewers**

**Manuscript PONE-D-25-66618**

**Original title:** *Trends in Antimicrobial Resistance Among MRSA and MSSA Clinical Isolates: A Retrospective Analysis from 2013 to 2024 in Saudi Arabia*

**Revised title:** *Methicillin resistance and antimicrobial non-susceptibility trends among Staphylococcus aureus clinical isolates in Aseer, Saudi Arabia (2013–2024): a retrospective laboratory-based surveillance study*

We thank the academic editor and the three reviewers for their thorough and constructive comments, which have substantially improved the manuscript. Our point-by-point responses are set out below. Reviewer comments are shown in italics, each followed by our response. Unless stated otherwise, all changes are visible in the marked-up (tracked-changes) file, and section references are to the revised manuscript.

**Response to Editorial and Journal Requirements**

In addition to the reviewers’ comments, we have addressed the journal requirements set out in the decision letter. The amended Funding Statement, the Role of Funder statement, and the author-support statement are provided in full in the accompanying Cover Letter for entry into the online submission form, and are summarised here so that this response is self-contained.

**1. Style and file naming.** The manuscript has been formatted in accordance with the PLOS ONE style templates, and all files have been named as requested (Manuscript; Revised Manuscript with Track Changes; Response to Reviewers; Cover Letter; and the figure files).

**2. Funding text in the manuscript.** All funding-related text has been removed from the Acknowledgments section and from the body of the manuscript. The full Financial Disclosure / Funding Statement (“This work was supported by the Deanship of Research and Graduate Studies at King Khalid University (https://www.kku.edu.sa) through the Small Research Project programme, grant number RGP1/289/46, awarded to AJA; the funders had no role in study design, data collection and analysis, decision to publish, or preparation of the manuscript”) is provided in the Cover Letter for the editorial office to enter in the submission form.

**3. Role of the funder.** The funder had no role in the study. The following statement is provided in the Cover Letter: “The funders had no role in study design, data collection and analysis, decision to publish, or preparation of the manuscript.”

**4. Author affiliation with the funder.** Several authors (AJA, YS, AS, IMA, AAB and MMA) are affiliated with King Khalid University, the funding organisation. As set out in the Cover Letter, the funder’s support for this study took the form of research grant RGP1/289/46 and did not include author salaries; the funder had no role in the study design, data collection and analysis, decision to publish, or preparation of the manuscript, and the specific roles of all authors are described in the Author Contributions section.

**5. Ethics statement.** The ethics statement now appears only in the Methods section (Study Design and Population) and has been removed from all other sections. It states that the study was approved by the Aseer Institutional Review Board, Aseer Branch, Ministry of Health, Saudi Arabia (approval F7-2-2025, dated 16 April 2025), with a waiver of informed consent for this retrospective analysis of routinely collected, de-identified laboratory data.

**6. Reviewer-recommended citations.** We reviewed the works suggested by the reviewers, removed the citations identified as irrelevant or misinterpreted, and added appropriate, peer-reviewed and regionally relevant references where they strengthen the manuscript.

**ORCID.** All co-authors will verify their ORCID iDs within the submission system before final acceptance, as only the individual author can complete this step.

**Figures.** The figures are provided within the manuscript; if separate figure files are required at resubmission, they will be prepared to meet the PLOS ONE technical specifications.

**Academic Editor**

**Comment.** *Revise the whole manuscript in light of all reviewers’ comments and submit the revised version.*

**Response.** We have revised the entire manuscript in response to all reviewer comments, as detailed below. The title, abstract, introduction, methods, results, discussion, conclusions, declarations, and references have all been revised.

**Reviewer #1**

**Title.** *The title is too long and academically clichéd. The study is limited to a single tertiary-care centre in Saudi Arabia, so extending the title to cover the whole country is incorrect. Please rephrase it to be more scientifically precise and to indicate the specific region surveyed.*

**Response.** We agree. The title has been rewritten to identify the study as single-centre surveillance in the Aseer region and to reflect the design and outcomes accurately (see the revised title above). A short running title has also been added.

**Abstract.** *The abstract has typographical errors and applies inappropriate statistical methods to a large longitudinal dataset. The Results should give actual percentages, proportions and confidence intervals for MRSA and MSSA, the numbers of MRSA and MSSA isolates, their distribution among inpatients and outpatients, and p-values, odds ratios or chi-square values for the comparisons described as significant. The description “retrospective cross-sectional study” is incorrect for a study spanning more than a decade. The concluding sentence is too generic.*

**Response.** The abstract has been rewritten. It now reports the number and proportion of MRSA (2,231; 53.2%, 95% CI 51.7–54.7) and MSSA (1,963; 46.8%) isolates, their distribution by setting (for example, 1,975 of 2,231 MRSA isolates [88.5%] from inpatients), the temporal change in the MRSA share (47.4% in 2013 to 60.4% in 2024) with the adjusted odds ratio per calendar year (1.09, 95% CI 1.06–1.12), and the between-group non-susceptibility comparisons with false-discovery-rate-adjusted significance. The study is now described as a retrospective laboratory-based surveillance study, the conclusions are specific to the Aseer findings, and the typographical errors have been corrected.

**Keywords.** *The keyword list is generic and repeats terms from the title and abstract, which reduces discoverability.*

**Response.** The keywords have been replaced with terms that do not duplicate the title, to improve indexing: bloodstream infection; clindamycin resistance; empirical therapy; fluoroquinolone resistance; fusidic acid resistance; linezolid susceptibility; skin and soft tissue infection; vancomycin susceptibility.

**Introduction (colonisation).** *The claim that the bacterium colonises 25–30% of the population at any given time is not correct, because nasal carriage follows distinct patterns of persistent, intermittent and non-carriage. Please rephrase.*

**Response.** The sentence has been rewritten to describe carriage in terms of persistent carriers, intermittent carriers and non-carriers, rather than a single fixed proportion, and the colonisation figure has been removed.

**Introduction (pathogenicity versus virulence).** *The terms “pathogenicity” and “virulence” are used interchangeably, which is incorrect; the toxins and surface proteins described relate to virulence, not pathogenicity. Anthropomorphic or militaristic terms such as “arsenal” should be replaced with objective terms.*

**Response.** We have corrected the terminology. The text now states that the organism’s capacity to cause disease (pathogenicity) is the net result of multiple virulence determinants, and the word “arsenal” and similar language have been removed.

**Introduction (mechanism versus clinical concern).** *The account of MRSA is reductive (a “one gene, one phenotype” view). The clinical threat of MRSA is not due exclusively to PBP2a; it reflects a complex interplay of factors. The mechanism of resistance should be separated from the reasons it is a clinical concern.*

**Response.** This passage has been rewritten. It now distinguishes the molecular mechanism of methicillin resistance (acquisition of mecA, or its homologue mecC, encoding PBP2a) from the clinical and public-health importance of MRSA, which we attribute to the combination of β-lactam resistance, frequent co-resistance to other classes, and the organism’s virulence and transmissibility.

**Introduction (WHO and referencing errors).** *Make the clinical and geographic relevance clearer and correct the referencing errors. Reference 7 (the WHO “dirty dozen”, a 2017 news article) is obsolete and not peer-reviewed, and reference 8 (an FAO manual on data disaggregation) is unrelated to clinical microbiology.*

**Response.** This section has been rewritten to add national and regional context for Saudi Arabia. The WHO “dirty dozen” news item has been replaced with the peer-reviewed WHO priority-pathogens article (Tacconelli et al., Lancet Infect Dis 2018), and the FAO manual has been replaced with the Global Burden of Bacterial Antimicrobial Resistance study (Antimicrobial Resistance Collaborators, Lancet 2022).

**Introduction (MSSA context and “resistance rates”).** *A comparison between MRSA and MSSA needs a sound clinical context; clarify what MSSA represents and its significance for Saudi Arabia and the wider MENA region. The term “resistance rates” is epidemiologically inaccurate because it requires a time denominator.*

**Response.** We have added clinical context, noting that MSSA remains a major cause of serious infection and is generally treated with β-lactams, so distinguishing the two organisms and monitoring both is clinically important. Throughout the manuscript we have replaced “resistance rates” with “non-susceptibility” or “non-susceptibility proportion”, reserving rate-based language for the time-denominated trend analyses.

**Introduction (questionable citations).** *Alghoribi et al. (2020) concerns OXA-48 carbapenemase-producing Salmonella enterica and is unrelated to S. aureus, and Alhazmi et al. (2025) does not report a 30–50% prevalence of S. aureus.*

**Response.** We have removed the Alghoribi et al. (OXA-48 Salmonella) citation, which was cited in error, and corrected the prevalence statement. The manuscript now reports that MRSA prevalence across Saudi centres has been described at approximately 9–17%, with community-associated MRSA in roughly 23–42% of isolates in some regions, citing appropriate Saudi sources, and no longer attributes a 30–50% figure to Alhazmi et al.

**Introduction (novelty).** *The claim is factually incorrect and ignores recent large-scale retrospective cohort studies in Saudi Arabia; the HA-MRSA versus CA-MRSA distinction has been studied in the region. Justify the novelty of the work in light of the available high-quality surveillance data.*

**Response.** We have rewritten this passage to acknowledge the existing Saudi surveillance literature, including multicentre and regional reports and studies of HA-MRSA and CA-MRSA. We now frame the contribution more precisely: the simultaneous, longitudinal tracking of non-susceptibility in both MRSA and MSSA, across inpatient and outpatient settings, in the Aseer region specifically, where such longitudinal data are limited.

**Introduction (objectives).** *The stated objectives are redundant, because local treatment guidelines and stewardship programmes are well established in Saudi healthcare; the study should not be framed as devising empirical strategies from scratch.*

**Response.** We agree and have reframed the objectives. The manuscript now states explicitly that Saudi facilities already operate stewardship programmes and national guidance, and that the aim is not to devise empirical guidance de novo but to provide updated, locally specific evidence to support and refine it. A reference to Saudi stewardship programmes (Alghamdi et al., Antibiotics 2021) has been added.

**Methods (exclusions, data access, authorship).** *Nearly 20% of the cohort (932 paediatric and neonatal isolates) was excluded without justification, introducing selection bias; it is implausible that a decade of data could be accessed in a single day; and the claim that patient details were not revealed is inconsistent with having identified and excluded paediatric and neonatal samples. The roles of the researchers in data extraction, cleaning and analysis should be defined in line with ICMJE requirements.*

**Response.** We have substantially rewritten this section and clarified the cohort derivation. The laboratory information system export used for the final analysis contained 4,194 S. aureus isolates from 3,122 unique patients aged 12 years or older; no records from patients younger than 12 years were present in the analytic dataset, so no paediatric or neonatal isolates were excluded during data cleaning. From 2016 onward the hospital did not routinely provide care for patients younger than 12 years, which explains the absence of such records in the later period. The medical record number was used only to link repeated isolates from the same patient and was removed before analysis; no other identifying information was accessed. Author roles in conceptualisation, data curation, analysis and writing are now specified in a CRediT-format Author Contributions section, consistent with ICMJE guidance.

**Methods (platforms, breakpoints, molecular testing).** *Using two automated platforms (BD Phoenix 100 and VITEK 2) interchangeably over the study period is a concern because they use different detection principles, and CLSI breakpoints were revised several times during 2013–2024. The Xpert MRSA NxG assay cannot perform SCCmec chromosomal typing; it is a diagnostic PCR kit. It is unclear how 100 MRSA isolates were “randomly selected” without knowing the total, and their distribution across the study period and by setting should be stated.*

**Response.** We have clarified the microbiological methods. Both platforms were used in parallel and interchangeably according to availability during routine service, with no single transition date, and susceptibility results were interpreted according to the contemporaneous CLSI M100 breakpoints in use at the time of testing (23rd through 34th editions). We have corrected the description of the molecular assay: the Xpert MRSA NxG assay detects MRSA-associated targets, including mecA and the SCCmec–orfX junction, and does not perform SCCmec typing. We now state that 100 phenotypically defined MRSA isolates from the final two surveillance years, all from inpatients, were tested after the assay was introduced, that this served as a confirmatory quality-assurance subset rather than a typing or epidemiological exercise, and that it is not representative of the full study period. This limitation is now discussed explicitly.

**Statistical analysis.** *The statistical analysis is unsuitable for a long-term, large-scale retrospective study. The use of Fisher’s exact test for “small expected cell counts” is implausible given the large sample. Reducing a decade of data to cumulative frequencies masks the resistance trends that were the goal of the study, and univariate comparisons do not account for confounding.*

**Response.** We have rebuilt the statistical analysis. Annual non-susceptibility proportions are reported with Wilson 95% confidence intervals, and temporal trends are quantified by segmented log-linear (joinpoint) regression with the annual percent change, computed for all S. aureus and within the MRSA and MSSA strata. Between-group comparisons use the Pearson chi-square test, with the Fisher exact test reserved for instances where an expected cell count is below five, and are additionally adjusted for multiple comparisons using the Benjamini–Hochberg false discovery rate. Independent predictors of MRSA are estimated with a multivariable generalised estimating equation (GEE) logistic regression that adjusts for calendar year, age group, sex, setting, ward, source and diagnosis, and accounts for repeated isolates from the same patient. De-duplicated sensitivity analyses are also reported. These additions directly address the trend-analysis, confounding and multiple-comparison concerns.

**Reviewer #2**

**Comment 1 (Abstract).** *Briefly add existing literature on the topic.*

**Response.** The abstract now situates the study within the existing literature, noting that MRSA is a high-priority resistance threat and that MRSA and MSSA differ in non-β-lactam resistance, while highlighting the limited longitudinal data from the Aseer region.

**Comment 2 (Background).** *Briefly mention the cause of MRSA’s resistance to non-β-lactam antibiotics.*

**Response.** The introduction now explains that non-β-lactam resistance arises in part because the mobile elements carrying mecA may also carry additional resistance determinants and because resistant lineages accumulate further mutations, and it lists the relevant mechanisms (enzymatic drug modification, ribosomal target modification, target-site mutation and active efflux).

**Comment 3 (Microbiological methods).** *Provide the reference to the exact CLSI guidelines used to interpret susceptibility.*

**Response.** We now specify that susceptibility was interpreted according to the contemporaneous CLSI M100 breakpoints in use at the time of testing (23rd through 34th editions, 2013–2024) and have added the corresponding CLSI reference to the reference list.

**Comment 4 (Results).** *If antimicrobial resistance was analysed separately by specimen type, mention it.*

**Response.** We have added a subsection on the distribution by setting, ward category and source category, and we report source-stratified MRSA proportions in a supplementary table.

**Comment 5 (Discussion).** *Compare your results with existing literature in terms of percentages where available, and discuss the efficacy of susceptible antimicrobials with respect to the site of infection.*

**Response.** The discussion now compares our non-susceptibility values with a recent United States outpatient series and discusses oral options for skin and soft-tissue infection (trimethoprim–sulfamethoxazole, tetracyclines and clindamycin), while noting that empirical choices should be guided by local susceptibility data and that vancomycin and daptomycin retained full in vitro activity.

**Comment 6 (Limitations).** *Mention other limitations, such as the fact that the linezolid response was not evaluated.*

**Response.** The limitations now state that confirmatory testing for linezolid was not performed and that the low-level linezolid signal is therefore unconfirmed, and they additionally note the unavailability of MIC distributions, the combined non-susceptible category, the use of two platforms and successive breakpoint revisions, the single-centre design, and the retention of repeated isolates in the primary analysis.

**Reviewer #3**

**Major concerns**

**Study duration inconsistency.** *The manuscript refers to an 11-year period in the title and abstract, to January 2013–June 2024, and to a 12-year period in the Methods. The duration should be described consistently.*

**Response.** We have standardised the description throughout. The study period is now described consistently as January 2013 to June 2024, and the inconsistent “11-year” and “12-year” references have been removed.

**“Trends” claim.** *The title emphasises trends, but only pooled resistance data are presented; no year-wise trends or trend statistics are given. Either include temporal analyses or revise the title.*

**Response.** We have done both. The manuscript now presents the annual distribution of MRSA and MSSA (Table 2, Figure 1), formal temporal trend analyses by segmented log-linear regression with the annual percent change (Table 3, Figure 2), and era-based trends with a p-for-trend (Supplementary Table S5), and the title has been revised to reflect the design and findings accurately.

**Potential inconsistency in Table 3.** *Table 3 reports 2,231 MRSA isolates stratified by setting; please verify that sample sizes, percentages and narrative descriptions are internally consistent, as any discrepancy could affect the interpretation of erythromycin resistance.*

**Response.** The tables have been rebuilt and audited for internal consistency. MRSA totals (n=2,231; inpatients 1,975, outpatients 256) are now reported consistently across the baseline table, the text and the analyses, and the setting-related findings are presented within the trend and era analyses.

**Limited molecular characterisation.** *Only 100 MRSA isolates were confirmed by GeneXpert; the rationale, sampling strategy and representativeness should be clarified and discussed.*

**Response.** We have clarified that the 100 isolates were drawn from the final two surveillance years and were all from inpatients, that they were tested after the assay became available, and that the subset served as a confirmatory quality-assurance check rather than a representative or typing exercise. This is now stated in the Methods and discussed as a limitation.

**Statistical comments**

**Table 1 statistical testing.** *Footnotes refer to both chi-square and Fisher’s exact tests; indicate which test was applied to each comparison.*

**Response.** The baseline-table footnote now specifies the test used for each variable: the Mann–Whitney U test for age, and the chi-square test, or the Fisher exact test when any expected cell count was below five, for the categorical variables.

**Multiple comparisons.** *Indicate whether adjustment for multiple testing was considered, or justify not doing so.*

**Response.** We now adjust the antibiotic-specific MRSA-versus-MSSA comparisons for multiple testing using the Benjamini–Hochberg false discovery rate, and we report the adjusted q-values alongside the p-values (Table 4).

**Confidence intervals.** *Reporting 95% confidence intervals alongside resistance estimates would improve interpretability.*

**Response.** Annual MRSA proportions are now reported with Wilson 95% confidence intervals (Table 2), and the overall MRSA proportion is reported with its confidence interval.

**Data availability**

**Comment.** *Provision of supplementary datasets or summary tables would support transparency and reproducibility.*

**Response.** We have added supplementary tables (cohort derivation; setting- and source-stratified distributions; full tested denominators with susceptible and non-susceptible counts; era-based trends; and de-duplicated sensitivity analyses) and a data-availability statement describing the conditions for access to the de-identified data.

**Language and formatting**

**Comment.** *Examples include: “Recent statistic” (line 90) should be “Recent statistics”; lines 119–121 require revision; “all hospital sitting” (line 133) should be “all hospital settings”; and p-values should be formatted consistently.*

**Response.** These errors have been corrected. The passages in question have been rewritten, the noted phrases no longer appear, and p-values are now formatted consistently throughout the manuscript.

**Minor revisions**

**Abstract Methods fragment.** *“A retrospective cross-sectional study to investigate and compare the antimicrobial resistance patterns…” should be a complete sentence.*

**Response.** The Methods sentence in the abstract is now complete; it begins, “We conducted a retrospective laboratory-based surveillance study of 4,194 S. aureus isolates…”.

**Reference verification (reference 11).** *Reference 11 appears to concern Salmonella enterica serovar Kentucky and does not support the statement on MRSA prevalence; please verify and replace.*

**Response.** The Salmonella enterica (OXA-48) reference has been removed, and the reference list has been re-checked and reformatted in the journal style.

**Vancomycin and daptomycin susceptibility.** *Inclusion of MIC distributions or MIC trend data would strengthen the surveillance value.*

**Response.** No vancomycin or daptomycin non-susceptibility was detected, and these agents are reported descriptively. MIC distributions were not captured in the laboratory dataset, which is now stated as a limitation; we agree that prospective MIC surveillance would add value and have noted this as a direction for future work.

**Discussion (speculative interpretations).** *Some interpretations regarding community prescribing and demographic explanations are speculative and should be presented more cautiously.*

**Response.** We have removed or substantially tempered these interpretations. The discussion now states explicitly that, as surveillance data without linked antimicrobial-consumption information, the dataset cannot establish causal or community-versus-hospital attributions, and the demographic interpretations have been softened to hypothesis-generating statements or removed.

We thank the editor and the reviewers once again for their time and for comments that have meaningfully strengthened the manuscript.
