# Supplementary material for: Methicillin resistance and antimicrobial non-susceptibility trends among Staphylococcus aureus clinical isolates in Aseer, Saudi Arabia (2013–2024): A retrospective laboratory-based surveillance study
Source: PLoS One. 2026 Sep 15;21(9):e0357789. doi: 10.1371/journal.pone.0357789 (PMC13577330; doi:10.1371/journal.pone.0357789)
Supplement: Peer review history

# Peer review: Decision Letter 0

**DOI:** 10.1371/journal.pone.0357789.r001

**Authors:** Samiullah Khan

8 Jun 2026

Please submit your revised manuscript by Jul 23 2026 11:59PM. If you will need more time than this to complete your revisions, please reply to this message or contact the journal office at plosone@plos.org. When you're ready to submit your revision, log on to https://www.editorialmanager.com/pone/ and select the 'Submissions Needing Revision' folder to locate your manuscript file.

- A letter that responds to each point raised by the academic editor and reviewer(s). You should upload this letter as a separate file labeled 'Response to Reviewers'.
- A marked-up copy of your manuscript that highlights changes made to the original version. You should upload this as a separate file labeled 'Revised Manuscript with Track Changes'.
- An unmarked version of your revised paper without tracked changes. You should upload this as a separate file labeled 'Manuscript'.

If you would like to make changes to your financial disclosure, please include your updated statement in your cover letter. Guidelines for resubmitting your figure files are available below the reviewer comments at the end of this letter.

If applicable, we recommend that you deposit your laboratory protocols in protocols.io to enhance the reproducibility of your results. Protocols.io assigns your protocol its own identifier (DOI) so that it can be cited independently in the future. For instructions see: https://journals.plos.org/plosone/s/submission-guidelines#loc-laboratory-protocols. Additionally, PLOS ONE offers an option for publishing peer-reviewed Lab Protocol articles, which describe protocols hosted on protocols.io. Read more information on sharing protocols at https://plos.org/protocols?utm_medium=editorial-email&utm_source=authorletters&utm_campaign=protocols.

As the corresponding author, your ORCID iD is verified in the submission system and will appear in the published article. PLOS supports the use of ORCID, and we encourage all coauthors to register for an ORCID iD and use it as well. Please encourage your coauthors to verify their ORCID iD within the submission system before final acceptance, as unverified ORCID iDs will not appear in the published article. *Only*  the individual author can complete the verification step; PLOS staff *cannot*  verify ORCID iDs on behalf of authors.

We look forward to receiving your revised manuscript.

Kind regards,

Samiullah Khan, Ph. D

Academic Editor

PLOS One

Journal Requirements:

When submitting your revision, we need you to address these additional requirements.

1. Please ensure that your manuscript meets PLOS ONE's style requirements, including those for file naming. The PLOS ONE style templates can be found at

https://journals.plos.org/plosone/s/file?id=wjVg/PLOSOne_formatting_sample_main_body.pdf and

https://journals.plos.org/plosone/s/file?id=ba62/PLOSOne_formatting_sample_title_authors_affiliations.pdf

2. Thank you for stating the following in the Acknowledgments Section of your manuscript:

“The authors extend their appreciation to the Deanship of Research and Graduate Studies at King Khalid University for funding this work through Small Research Project under grant number RGP1/289/46”

We note that you have provided funding information that is not currently declared in your Funding Statement. However, funding information should not appear in the Acknowledgments section or other areas of your manuscript. We will only publish funding information present in the Funding Statement section of the online submission form.

Please remove any funding-related text from the manuscript and let us know how you would like to update your Funding Statement. Currently, your Funding Statement reads as follows:

“Deanship of Research and Graduate Studies at King Khalid University for funding this work through Small Research Project under grant number RGP1/289/46”

Please include your amended statements within your cover letter; we will change the online submission form on your behalf.

3. Thank you for stating the following financial disclosure:

“Deanship of Research and Graduate Studies at King Khalid University for funding this work through Small Research Project under grant number RGP1/289/46”

Please state what role the funders took in the study.  If the funders had no role, please state: "The funders had no role in study design, data collection and analysis, decision to publish, or preparation of the manuscript."

If this statement is not correct you must amend it as needed.

Please include this amended Role of Funder statement in your cover letter; we will change the online submission form on your behalf.

4. Thank you for stating the following financial disclosure:

“Deanship of Research and Graduate Studies at King Khalid University for funding this work through Small Research Project under grant number RGP1/289/46”

We note that one or more of the authors is affiliated with the funding organization, indicating the funder may have had some role in the design, data collection, analysis or preparation of your manuscript for publication; in other words, the funder played an indirect role through the participation of the co-authors. If the funding organization did not play a role in the study design, data collection and analysis, decision to publish, or preparation of the manuscript and only provided financial support in the form of authors' salaries and/or research materials, please do the following:

1. Review your statements relating to the author contributions, and ensure you have specifically and accurately indicated the role(s) that these authors had in your study. These amendments should be made in the online form.

2. Confirm in your cover letter that you agree with the following statement, and we will change the online submission form on your behalf:

“The funder provided support in the form of salaries for authors [insert relevant initials], but did not have any additional role in the study design, data collection and analysis, decision to publish, or preparation of the manuscript. The specific roles of these authors are articulated in the ‘author contributions’ section.

5. Your ethics statement should only appear in the Methods section of your manuscript. If your ethics statement is written in any section besides the Methods, please move it to the Methods section and delete it from any other section. Please ensure that your ethics statement is included in your manuscript, as the ethics statement entered into the online submission form will not be published alongside your manuscript.

6. If the reviewer comments include a recommendation to cite specific previously published works, please review and evaluate these publications to determine whether they are relevant and should be cited. There is no requirement to cite these works unless the editor has indicated otherwise.

Additional Editor Comments:

Dear author

Revise the whole manuscript in light of all reviewer's comments and submit the revised version.

[Note: HTML markup is below. Please do not edit.]

Reviewers' comments:

Reviewer's Responses to Questions

**Comments to the Author**

1. Is the manuscript technically sound, and do the data support the conclusions?

Reviewer #1: No

Reviewer #2: Yes

Reviewer #3: Partly

**********

2. Has the statistical analysis been performed appropriately and rigorously?

Reviewer #1: No

Reviewer #2: Yes

Reviewer #3: No

**********

3. Have the authors made all data underlying the findings in their manuscript fully available?

The PLOS Data policy

Reviewer #1: Yes

Reviewer #2: Yes

Reviewer #3: Yes

**********

4. Is the manuscript presented in an intelligible fashion and written in standard English?

Reviewer #1: No

Reviewer #2: Yes

Reviewer #3: Yes

**********

Reviewer #1: Title:

ln 1-3; is too long and academically clichéd. The study is limited to a single tertiary care center in KSA; therefore, extending the title to cover all of Saudi Arabia is incorrect. Rephrase the title to be more scientifically precise and to indicate the specific regions of KSA it surveils.

Abstract

ln 45-68, the abstract has several typographical errors and methodological issues, especially the inappropriate application of statistical methods to a large longitudinal dataset. A thorough revision is therefore required. The "Results" section should not be vague; instead, it should provide actual percentages, proportions, or confidence intervals for the MRSA and MSSA groups. How many of the total isolates in the samples were MRSA, and how many MSSA? Their distribution among inpatients and outpatients is also not given. The abstract should provide p-values, odds ratios or chi-square metrics for the comparisons in which the authors claim to find "significant differences" in antibiotic profiles. The authors also describe their work as a "retrospective cross-sectional study," but a study that spans more than a decade of tracking shifts in microbial AMR profiles is a longitudinal surveillance or a retrospective cohort study. I think the author's style of concluding by stating that "Continuous surveillance and tailored antimicrobial stewardship are imperative" is too generic and banal. It can be made meaningful if it discusses specific findings from the Aseer region data, local resistance trends and their impact on clinical treatments.

Keywords

ln 69; The current list is generic, which does not add value to the search engine optimization (SEO) of the paper. Terms in the title and abstract should be avoided, as they reduce the manuscript's discoverability.

Introduction

ln 71-73; This statement needs to be rewritten for clarity and scientific accuracy. Too many concepts—including anatomy, statistics, and pathogenic potential have been lumped together into a single awkwardly made sentence. I don't think the authors are correct in stating that the bacteria colonize 25–30% of the population at any given time, since their prevalence in the nasal area fluctuates with distinct patterns of permanent, transient and non-carriers. Rephrase.

ln 78-80; the authors' tendency to use the terms 'pathogenicity' and 'virulence' interchangeably is also incorrect, since they describe distinct aspects of disease. The toxins and surface proteins the authors discuss relate to the bacterium's virulence, not its pathogenicity. The authors should also avoid terms with anthropomorphic or militaristic connotations, such as 'arsenal' for describing bacterial aspects, and replace them with objective, academic terms.

ln 83-85; I think the narrative here is grossly reductive, reminiscent of the 'one gene, one phenotype' paradigm in the context of the clinical challenge of MRSA. A biochemical explanation of resistance does not account for the pathogen's epidemiological success or clinical virulence. It would be incorrect to state that the threat of MRSA is exclusively due to the presence of PBP2a; it is the manifestation of a complex interplay of several virulence factors. The authors ought to separate how bacteria gain resistance from how this is a clinical concern, against a backdrop of the genomics of MRSA pathogenesis.

ln 87-91; this section should be completely revamped to make the clinical and geographic relevance clearer, as well as to correct referencing errors. I don't think the authors should rely on generalities like the global WHO initiatives and ignore the national and regional contexts of KSA. There are several recent studies that the authors should cite to justify the scope of their study. Several cited references are academically below par for instance, reference # 7) Meet WHO's dirty dozen: The 12 bacteria for which new drugs are most urgently needed. AAAS Articles DO Group. 2021. doi:10.1126/science.aal0829

This is an obsolete 2017 science journalism news article and not a peer-reviewed scientific report! Similarly, reference #8) Guidelines on data disaggregation for SDG Indicators using survey data. FAO, 2021. 285 doi:10.4060/cb3253en is a manual published by FAO that has nothing to do with clinical microbiology! Can the authors justify including them here?

ln 92-94; "The authors have been abrupt in their use of terminology and regional context. A comparison between MRSA and MSSA requires a sound clinical context. The authors should clarify what MSSA represents and its clinical significance for KSA and the wider MENA region. The term 'resistance rates' is epidemiologically inaccurate, since it requires a time denominator, which is not the case here. Replace with a statistically accurate term.

ln 98-100; I think the authors have cited questionable studies to support their argument. The reference (Alghoribi et al., 2020) discusses OXA-48 carbapenemase-producing Salmonella enterica and has nothing to do with S. aureus or MRSA. The other reference (Alhazmi et al., 2025) has been grossly misinterpreted, as it does not report a 30–50% prevalence of S. aureus isolates anywhere. Either reanalyze or remove these citations.

ln 101-106, I think the authors' claim is factually incorrect, as it ignores several recent large-scale retrospective cohort studies conducted in KSA. Similarly, the distinction between HA-MRSA and CA-MRSA has been studied in this region. The authors must provide an accurate justification for the novelty of their work in light of the high-quality surveillance data publicly available in KSA.

ln 107-110; in light of my above comment, the authors' stated objectives are also redundant. Local treatment guidelines, antibiotic protocols for MRSA and MSSA are well established in Saudi healthcare facilities. One cannot claim that its objective is to 'recognize appropriate empiric choices' when the country has functional stewardship programs. The authors should refrain from framing it as a pioneering study for making empirical treatment strategies from scratch. The following are examples of recent works on antibiotic stewardship in KSA, which can help the authors frame their objectives.

1. Al-Oman A, et al. Antimicrobial Stewardship Programs in Saudi Hospitals. Antibiotics (Basel). 2021;10(2):193. doi:10.3390/antibiotics10020193.

2. Al-Garni MA, et al. Antimicrobial utilization among hospitalized patients according to WHO AWaRe Classification: results from a multicentre point prevalence survey in Saudi Arabia. Journal of Global Antimicrobial Resistance. 2025/2026. doi:10.1016/j.jgar.2025.xx.xxx.

Methods

ln 113-121; The authors dropped 932 pediatric and neonatal isolates (nearly 20% of the cohort) without providing any scientific justification. This exclusion introduces severe selection bias and degrades the value of this data for developing evidence-based clinical options. I find the authors' claim implausible that they were able to access a decade's worth of lab data in a single day, which brings to light issues like data integrity and quality control. I also find the author's claim tenuous that 'patient details were not revealed to the research team while in the same breath also claiming to have identified and excluded 932 pediatric and neonatal samples! Surely the authors cannot be ignorant of the particulars of these patients since they used age-based demographic criteria for screening. The detached reference to 'the research team' is also a concern, as it raises issues of authorship and data ownership. The exact roles of the researchers in the data extraction, cleaning, and analysis processes should be clearly defined in line with ICMJE authorship requirements.

ln 123-133; I think the use of two completely different automated platforms—BD Phoenix 100 and VITEK 2—interchangeably across an 11-year study period is a serious concern. These platforms use different detection principles and proprietary algorithms to calculate MICs. Additionally, the breakpoints specified by CLSI have undergone multiple revisions during the timeframe of 2013 to 2024, so how this 'methodological consistency and comparability were ensured' should be made clear! The authors' claim of using the Xpert® MRSA NxG kit on the GeneXpert® system to confirm the SCCmec gene is also technically flawed, as this system cannot perform SCCmec chromosomal typing. It is a basic diagnostic PCR kit and not a molecular typing tool. It is unclear to me when the authors state they 'randomly selected' 100 MRSA isolates for molecular testing. How did they evaluate the scientific validity of this sample without knowing the total number of MRSA isolates it was drawn from? The distribution of these 100 isolates across the 12-year study period and across inpatient/outpatient categories should also be made clear.

ln 134-140; I find the 'Statistical Analysis' subsection grossly unsuitable for a long-term, large-scale retrospective epidemiological study. The authors state they used Fisher's exact test due to 'the presence of small, expected cell counts,' which is mathematically infeasible given the large sample size and the fact that Fisher's exact test is meant for small-sample contingency tables, not for thousands of points across demographic data. Also, reducing a decade of dynamic epidemiological data into cumulative frequencies masks tracking of resistance trends over time, which was the goal of this study. The dataset contains multiple clinical and demographic variables, and univariate cell comparisons do not account for confounding, a glaring deficiency.

Reviewer #2: My analysis of this research study: This is a cross-sectional study aimed to evaluate and compare the antimicrobial resistance pattern for MRSA and MSSA, in outpatient and inpatient settings. Results showed increased resistance to antimicrobials including macrolides, flouroquinolones, aminogylcosides among MRSA as compared to MSSA. No resistance to vancomycin or daptomycin found.

COMMENTS:

1.Abstract page 3 and 4: Briefly add existing literature on your topic.

2. Background page 5: Briefly mention the cause of MRSA's resistance to non lactam antibiotics.

3.Microbiological methods page 7: Please provide the reference to exact CLSI guidelines used to interpret susceptibility.

4. Results page 12: If antimicrobial resistance pattern was analyzed separately by specimen type, mention here.

5.Discussion page 13: Compare your results with existing literature regarding antimicrobials' resistance in terms of percentages if available.

Discuss the efficacy of susceptible antimicrobials with respect to site of infection.

6.Limitations page 14: Mention the other limitations like linezolid response was not evaluated.

Reviewer #3: This manuscript addresses an important public health issue and presents a substantial dataset comprising 4,194 non-duplicate Staphylococcus aureus isolates collected over more than a decade. The study provides valuable local antimicrobial resistance surveillance data and may contribute to antimicrobial stewardship efforts in the region. However, several methodological and reporting issues require clarification before the conclusions can be fully supported.

1. Major Concerns

Study duration inconsistency

The manuscript refers to an 11-year study period in the title and abstract, whereas the study period extends from January 2013 to June 2024. In addition, the Microbiological Methods section refers to a 12-year study period. The study duration should be described consistently throughout the manuscript.

Title emphasizes "trends" but trend analyses are not presented

The title highlights antimicrobial resistance trends from 2013–2024; however, the results present pooled resistance data across the entire study period. No year-wise resistance trends, temporal analyses, or trend statistics are provided. The authors should either include appropriate temporal trend analyses or revise the title to better reflect the study design and findings.

Potential inconsistency in Table 3

Table 3 reports a total of 2,231 MRSA isolates, stratified into inpatient and outpatient groups. The authors should carefully verify that the sample sizes, percentages, and corresponding narrative descriptions are correctly labeled and internally consistent. Any discrepancy in the assignment of inpatient versus outpatient isolates could influence interpretation of the observed erythromycin resistance patterns and the subsequent discussion regarding community-associated resistance dynamics.

Limited molecular characterization

Only 100 MRSA isolates underwent molecular confirmation using GeneXpert. The rationale for selecting these isolates, the sampling strategy, and their representativeness of the overall MRSA population should be clarified. This limitation should also be discussed in greater detail.

2. The statistical approach is generally acceptable for categorical comparisons; however, several points require clarification.

Comments

Table 1 statistical testing

Table 1 contains footnotes referring to both Chi-square and Fisher’s exact tests. The manuscript should clearly indicate which statistical test was applied to each comparison.

Multiple comparisons

Numerous antibiotic-specific comparisons were performed across Tables 2 and 3. The authors should indicate whether adjustment for multiple testing was considered or provide justification for not performing such adjustment.

Confidence intervals

Reporting 95% confidence intervals alongside resistance estimates would improve interpretability and allow readers to assess the precision of estimates.

3. The authors indicate that all relevant data are contained within the manuscript. However, provision of supplementary datasets or summary tables would further support transparency and reproducibility in accordance with PLOS ONE data-sharing principles.

4. The manuscript is generally understandable and logically organized. However, several grammatical and formatting issues should be corrected.

Examples include:

• Line 90: “Recent statistic” should be revised to “Recent statistics.”

• Lines 119–121 require grammatical revision.

• Line 133: “all hospital sitting” should be revised to “all hospital settings.”

• Ensure consistent formatting of p-values throughout the manuscript.

5. Detailed Review Comments and Feedback

Major Revisions

1. Clarify study duration

The manuscript alternately describes the study as 11 years, 11.5 years, and 12 years. A single consistent description should be used throughout the manuscript.

2. Reconcile Table 3 and related interpretation

Please carefully audit Table 3 and the accompanying text to ensure that inpatient and outpatient sample sizes, percentages, resistance rates, and narrative descriptions are correctly reported and interpreted.

3. Address the “trends” claim

The title suggests temporal trend analysis, yet no annual trend data are presented. Either include trend analyses or revise the title accordingly.

4. Clarify molecular confirmation strategy

Please explain:

• Why only 100 isolates underwent molecular confirmation.

• How these isolates were selected.

• Whether they are representative of the broader MRSA collection.

5. Microbiological methodology

The manuscript reports use of both BD Phoenix and VITEK 2 systems during the study period. Please specify:

• When the transition occurred.

• Whether CLSI breakpoints changed during the study period.

• How methodological consistency was maintained across the surveillance period.

Minor Revisions

1. Abstract

The Methods section begins with the fragment:

“A retrospective cross-sectional study to investigate and compare the antimicrobial resistance patterns…”

This should be revised to a complete sentence.

2. Reference verification

Reference 11 appears to concern Salmonella enterica serovar Kentucky and does not appear to support the statement regarding MRSA prevalence in Saudi Arabia. Please verify and replace this citation if necessary.

3. Vancomycin and daptomycin susceptibility

No resistance was detected to vancomycin or daptomycin, which is reassuring. If available, inclusion of MIC distributions or MIC trend data would strengthen the surveillance value of the study.

4. Discussion

Some interpretations regarding community prescribing practices and demographic explanations are speculative and should be presented more cautiously unless supported by additional data.

Overall Assessment

This study addresses a clinically important topic and is strengthened by its large sample size and extended surveillance period. However, clarification of methodological details, verification of data presentation, resolution of title-versus-analysis inconsistencies, and correction of citation issues are necessary before the manuscript can be considered for publication.

**********

what does this mean?). If published, this will include your full peer review and any attached files.

If you choose “no”, your identity will remain anonymous but your review may still be made public.

**Do you want your identity to be public for this peer review?**  For information about this choice, including consent withdrawal, please see our Privacy Policy

Reviewer #1: No

Reviewer #2: **Yes:**  Fizza Khalid

Reviewer #3: **Yes:**  Dr. Godfred Antony Menezes

**********

[NOTE: If reviewer comments were submitted as an attachment file, they will be attached to this email and accessible via the submission site. Please log into your account, locate the manuscript record, and check for the action link "View Attachments". If this link does not appear, there are no attachment files.]

To ensure your figures meet our technical requirements, please review our figure guidelines: https://journals.plos.org/plosone/s/figures

You may also use PLOS’s free figure tool, NAAS, to help you prepare publication quality figures: https://journals.plos.org/plosone/s/figures#loc-tools-for-figure-preparation.

NAAS will assess whether your figures meet our technical requirements by comparing each figure against our figure specifications.

# Author comment: Author response to Decision Letter 1

**DOI:** 10.1371/journal.pone.0357789.r002

5 Jul 2026

Response to Editorial and Journal Requirements

In addition to the reviewers’ comments, we have addressed the journal requirements set out in the decision letter. The amended Funding Statement, the Role of Funder statement, and the author-support statement are provided in full in the accompanying Cover Letter for entry into the online submission form, and are summarised here so that this response is self-contained.

1. Style and file naming. The manuscript has been formatted in accordance with the PLOS ONE style templates, and all files have been named as requested (Manuscript; Revised Manuscript with Track Changes; Response to Reviewers; Cover Letter; and the figure files).

2. Funding text in the manuscript. All funding-related text has been removed from the Acknowledgments section and from the body of the manuscript. The full Financial Disclosure / Funding Statement (“This work was supported by the Deanship of Research and Graduate Studies at King Khalid University (https://www.kku.edu.sa) through the Small Research Project programme, grant number RGP1/289/46, awarded to AJA; the funders had no role in study design, data collection and analysis, decision to publish, or preparation of the manuscript”) is provided in the Cover Letter for the editorial office to enter in the submission form.

3. Role of the funder. The funder had no role in the study. The following statement is provided in the Cover Letter: “The funders had no role in study design, data collection and analysis, decision to publish, or preparation of the manuscript.”

4. Author affiliation with the funder. Several authors (AJA, YS, AS, IMA, AAB and MMA) are affiliated with King Khalid University, the funding organisation. As set out in the Cover Letter, the funder’s support for this study took the form of research grant RGP1/289/46 and did not include author salaries; the funder had no role in the study design, data collection and analysis, decision to publish, or preparation of the manuscript, and the specific roles of all authors are described in the Author Contributions section.

5. Ethics statement. The ethics statement now appears only in the Methods section (Study Design and Population) and has been removed from all other sections. It states that the study was approved by the Aseer Institutional Review Board, Aseer Branch, Ministry of Health, Saudi Arabia (approval F7-2-2025, dated 16 April 2025), with a waiver of informed consent for this retrospective analysis of routinely collected, de-identified laboratory data.

6. Reviewer-recommended citations. We reviewed the works suggested by the reviewers, removed the citations identified as irrelevant or misinterpreted, and added appropriate, peer-reviewed and regionally relevant references where they strengthen the manuscript.

ORCID. All co-authors will verify their ORCID iDs within the submission system before final acceptance, as only the individual author can complete this step.

Figures. The figures are provided within the manuscript; if separate figure files are required at resubmission, they will be prepared to meet the PLOS ONE technical specifications.

Academic Editor

Comment. Revise the whole manuscript in light of all reviewers’ comments and submit the revised version.

Response. We have revised the entire manuscript in response to all reviewer comments, as detailed below. The title, abstract, introduction, methods, results, discussion, conclusions, declarations, and references have all been revised.

Reviewer #1

Title. The title is too long and academically clichéd. The study is limited to a single tertiary-care centre in Saudi Arabia, so extending the title to cover the whole country is incorrect. Please rephrase it to be more scientifically precise and to indicate the specific region surveyed.

Response. We agree. The title has been rewritten to identify the study as single-centre surveillance in the Aseer region and to reflect the design and outcomes accurately (see the revised title above). A short running title has also been added.

Abstract. The abstract has typographical errors and applies inappropriate statistical methods to a large longitudinal dataset. The Results should give actual percentages, proportions and confidence intervals for MRSA and MSSA, the numbers of MRSA and MSSA isolates, their distribution among inpatients and outpatients, and p-values, odds ratios or chi-square values for the comparisons described as significant. The description “retrospective cross-sectional study” is incorrect for a study spanning more than a decade. The concluding sentence is too generic.

Response. The abstract has been rewritten. It now reports the number and proportion of MRSA (2,231; 53.2%, 95% CI 51.7–54.7) and MSSA (1,963; 46.8%) isolates, their distribution by setting (for example, 1,975 of 2,231 MRSA isolates [88.5%] from inpatients), the temporal change in the MRSA share (47.4% in 2013 to 60.4% in 2024) with the adjusted odds ratio per calendar year (1.09, 95% CI 1.06–1.12), and the between-group non-susceptibility comparisons with false-discovery-rate-adjusted significance. The study is now described as a retrospective laboratory-based surveillance study, the conclusions are specific to the Aseer findings, and the typographical errors have been corrected.

Keywords. The keyword list is generic and repeats terms from the title and abstract, which reduces discoverability.

Response. The keywords have been replaced with terms that do not duplicate the title, to improve indexing: bloodstream infection; clindamycin resistance; empirical therapy; fluoroquinolone resistance; fusidic acid resistance; linezolid susceptibility; skin and soft tissue infection; vancomycin susceptibility.

Introduction (colonisation). The claim that the bacterium colonises 25–30% of the population at any given time is not correct, because nasal carriage follows distinct patterns of persistent, intermittent and non-carriage. Please rephrase.

Response. The sentence has been rewritten to describe carriage in terms of persistent carriers, intermittent carriers and non-carriers, rather than a single fixed proportion, and the colonisation figure has been removed.

Introduction (pathogenicity versus virulence). The terms “pathogenicity” and “virulence” are used interchangeably, which is incorrect; the toxins and surface proteins described relate to virulence, not pathogenicity. Anthropomorphic or militaristic terms such as “arsenal” should be replaced with objective terms.

Response. We have corrected the terminology. The text now states that the organism’s capacity to cause disease (pathogenicity) is the net result of multiple virulence determinants, and the word “arsenal” and similar language have been removed.

Introduction (mechanism versus clinical concern). The account of MRSA is reductive (a “one gene, one phenotype” view). The clinical threat of MRSA is not due exclusively to PBP2a; it reflects a complex interplay of factors. The mechanism of resistance should be separated from the reasons it is a clinical concern.

Response. This passage has been rewritten. It now distinguishes the molecular mechanism of methicillin resistance (acquisition of mecA, or its homologue mecC, encoding PBP2a) from the clinical and public-health importance of MRSA, which we attribute to the combination of β-lactam resistance, frequent co-resistance to other classes, and the organism’s virulence and transmissibility.

Introduction (WHO and referencing errors). Make the clinical and geographic relevance clearer and correct the referencing errors. Reference 7 (the WHO “dirty dozen”, a 2017 news article) is obsolete and not peer-reviewed, and reference 8 (an FAO manual on data disaggregation) is unrelated to clinical microbiology.

Response. This section has been rewritten to add national and regional context for Saudi Arabia. The WHO “dirty dozen” news item has been replaced with the peer-reviewed WHO priority-pathogens article (Tacconelli et al., Lancet Infect Dis 2018), and the FAO manual has been replaced with the Global Burden of Bacterial Antimicrobial Resistance study (Antimicrobial Resistance Collaborators, Lancet 2022).

Introduction (MSSA context and “resistance rates”). A comparison between MRSA and MSSA needs a sound clinical context; clarify what MSSA represents and its significance for Saudi Arabia and the wider MENA region. The term “resistance rates” is epidemiologically inaccurate because it requires a time denominator.

Response. We have added clinical context, noting that MSSA remains a major cause of serious infection and is generally treated with β-lactams, so distinguishing the two organisms and monitoring both is clinically important. Throughout the manuscript we have replaced “resistance rates” with “non-susceptibility” or “non-susceptibility proportion”, reserving rate-based language for the time-denominated trend analyses.

Introduction (questionable citations). Alghoribi et al. (2020) concerns OXA-48 carbapenemase-producing Salmonella enterica and is unrelated to S. aureus, and Alhazmi et al. (2025) does not report a 30–50% prevalence of S. aureus.

Response. We have removed the Alghoribi et al. (OXA-48 Salmonella) citation, which was cited in error, and corrected the prevalence statement. The manuscript now reports that MRSA prevalence across Saudi centres has been described at approximately 9–17%, with community-associated MRSA in roughly 23–42% of isolates in some regions, citing appropriate Saudi sources, and no longer attributes a 30–50% figure to Alhazmi et al.

Introduction (novelty). The claim is factually incorrect and ignores recent large-scale retrospective cohort studies in Saudi Arabia; the HA-MRSA versus CA-MRSA distinction has been studied in the region. Justify the novelty of the work in light of the available high-quality surveillance data.

Response. We have rewritten this passage to acknowledge the existing Saudi surveillance literature, including multicentre and regional reports and studies of HA-MRSA and CA-MRSA. We now frame the contribution more precisely: the simultaneous, longitudinal tracking of non-susceptibility in both MRSA and MSSA, across inpatient and outpatient settings, in the Aseer region specifically, where such longitudinal data are limited.

Introduction (objectives). The stated objectives are redundant, because local treatment guidelines and stewardship programmes are well established in Saudi healthcare; the study should not be framed as devising empirical strategies from scratch.

Response. We agree and have reframed the objectives. The manuscript now states explicitly that Saudi facilities already operate stewardship programmes and national guidance, and that the aim is not to devise empirical guidance de novo but to provide updated, locally specific evidence to support and refine it. A reference to Saudi stewardship programmes (Alghamdi et al., Antibiotics 2021) has been added.

Methods (exclusions, data access, authorship). Nearly 20% of the cohort (932 paediatric and neonatal isolates) was excluded without justification, introducing selection bias; it is implausible that a decade of data could be accessed in a single day; and the claim that patient details were not revealed is inconsistent with having identified and excluded paediatric and neonatal samples. The roles of the researchers in data extraction, cleaning and analysis should be defined in line with ICMJE requirements.

Response. We have substantially rewritten this section and clarified the cohort derivation. The laboratory information system export used for the final analysis contained 4,194 S. aureus isolates from 3,122 unique patients aged 12 years or older; no records from patients younger than 12 years were present in the analytic dataset, so no paediatric or neonatal isolates were excluded during data cleaning. From 2016 onward the hospital did not routinely provide care for patients younger than 12 years, which explains the absence of such records in the later period. The medical record number was used only to link repeated isolates from the same patient and was removed before analysis; no other identifying information was accessed. Author roles in conceptualisation, data curation, analysis and writing are now specified in a CRediT-format Author Contributions section, consistent with ICMJE guidance.

Methods (platforms, breakpoints, molecular testing). Using two automated platforms (BD Phoenix 100 and VITEK 2) interchangeably over the study period is a concern because they use different detection principles, and CLSI breakpoints were revised several times during 2013–2024. The Xpert MRSA NxG assay cannot perform SCCmec chromosomal typing; it is a diagnostic PCR kit. It is unclear how 100 MRSA isolates were “randomly selected” without knowing the total, and their distribution across the study period and by setting should be stated.

Response. We have clarified the microbiological methods. Both platforms were used in parallel and interchangeably according to availability during routine service, with no single transition date, and susceptibility results were interpreted according to the contemporaneous CLSI M100 breakpoints in use at the time of testing (23rd through 34th editions). We have corrected the description of the molecular assay: the Xpert MRSA NxG assay detects MRSA-associated targets, including mecA and the SCCmec–orfX junction, and does not perform SCCmec typing. We now state that 100 phenotypically defined MRSA isolates from the final two surveillance years, all from inpatients, were tested after the assay was introduced, that this served as a confirmatory quality-assurance subset rather than a typing or epidemiological exercise, and that it is not representative of the full study period. This limitation is now discussed explicitly.

Statistical analysis. The statistical analysis is unsuitable for a long-term, large-scale retrospective study. The use of Fisher’s exact test for “small expected cell counts” is implausible given the large sample. Reducing a decade of data to cumulative frequencies masks the resistance trends that were the goal of the study, and univariate comparisons do not account for confounding.

Response. We have rebuilt the statistical analysis. Annual non-susceptibility proportions are reported with Wilson 95% confidence intervals, and temporal trends are quantified by segmented log-linear (joinpoint) regression with the annual percent change, computed for all S. aureus and within the MRSA and MSSA strata. Between-group comparisons use the Pearson chi-square test, with the Fisher exact test reserved for instances where an expected cell count is below five, and are additionally adjusted for multiple comparisons using the Benjamini–Hochberg false discovery rate. Independent predictors of MRSA are estimated with a multivariable generalised estimating equation (GEE) logistic regression that adjusts for calendar year, age group, sex, setting, ward, source and diagnosis, and accounts for repeated isolates from the same patient. De-duplicated sensitivity analyses are also reported. These additions directly address the trend-analysis, confounding and multiple-comparison concerns.

Reviewer #2

Comment 1 (Abstract). Briefly add existing literature on the topic.

Response. The abstract now situates the study within the existing literature, noting that MRSA is a high-priority resistance threat and that MRSA and MSSA differ in non-β-lactam resistance, while highlighting the limited longitudinal data from the Aseer region.

Comment 2 (Background). Briefly mention the cause of MRSA’s resistance to non-β-lactam antibiotics.

Response. The introduction now explains that non-β-lactam resistance arises in part because the mobile elements carrying mecA may also carry additional resistance determinants and because resistant lineages accumulate further mutations, and it lists the relevant mechanisms (enzymatic drug modification, ribosomal target modification, target-site mutation and active efflux).

Comment 3 (Microbiological methods). Provide the reference to the exact CLSI guidelines used to interpret susceptibility.

Response. We now specify that susceptibility was interpreted according to the contemporaneous CLSI M100 breakpoints in use at the time of testing (23rd through 34th editions, 2013–2024) and have added the corresponding

Attachment Submitted filename: Response to Reviewers.docx

# Peer review: Decision Letter 1

**DOI:** 10.1371/journal.pone.0357789.r003

**Authors:** Samiullah Khan

23 Aug 2026

Methicillin resistance and antimicrobial non-susceptibility trends among Staphylococcus aureus clinical isolates in Aseer, Saudi Arabia (2013–2024): a retrospective laboratory-based surveillance study

PONE-D-25-66618R1

Dear Dr. ,Abdulah J. Alqahtani, MD

We’re pleased to inform you that your manuscript has been judged scientifically suitable for publication and will be formally accepted for publication once it meets all outstanding technical requirements.

Within one week, you’ll receive an e-mail detailing the required amendments. When these have been addressed, you’ll receive a formal acceptance letter and your manuscript will be scheduled for publication.

An invoice will be generated when your article is formally accepted. Please note, if your institution has a publishing partnership with PLOS and your article meets the relevant criteria, all or part of your publication costs will be covered. Please make sure your user information is up-to-date by logging into Editorial Manager at Editorial Manager® and clicking the ‘Update My Information' link at the top of the page. For questions related to billing, please contact billing support.

If your institution or institutions have a press office, please notify them about your upcoming paper to help maximize its impact. If they’ll be preparing press materials, please inform our press team as soon as possible -- no later than 48 hours after receiving the formal acceptance. Your manuscript will remain under strict press embargo until 2 pm Eastern Time on the date of publication. For more information, please contact onepress@plos.org.

Kind regards,

Samiullah Khan, Ph. D

Academic Editor

PLOS One

Additional Editor Comments (optional):

Dear author

The reviewer is satisfied with all the changes made by author in revised manuscript, so I recommend that paper is now acceptable to be published in PLoS ONE.

Reviewers' comments:

Reviewer's Responses to Questions

**Comments to the Author**

Reviewer #4: All comments have been addressed

**********

2. Is the manuscript technically sound, and do the data support the conclusions?

Reviewer #4: Yes

**********

3. Has the statistical analysis been performed appropriately and rigorously?

Reviewer #4: Yes

**********

4. Have the authors made all data underlying the findings in their manuscript fully available?

The PLOS Data policy

Reviewer #4: Yes

**********

5. Is the manuscript presented in an intelligible fashion and written in standard English?

Reviewer #4: Yes

**********

Reviewer #4: The revised manuscript is substantially improved and addresses many of the concerns raised in the previous round. The title, abstract, methods, statistical analysis, and discussion are much clearer and more scientifically appropriate, and the longitudinal trend analysis adds genuine value. Please make a final editorial pass to correct minor formatting and terminology issues, including italicizing gene names (e.g. mecA and mecC) and S. aureus throughout.

**********

what does this mean?). If published, this will include your full peer review and any attached files.

If you choose “no”, your identity will remain anonymous but your review may still be made public.

**Do you want your identity to be public for this peer review?**  For information about this choice, including consent withdrawal, please see our Privacy Policy

Reviewer #4: No

**********

# Editor report: Acceptance letter

**DOI:** 10.1371/journal.pone.0357789.r004

**Authors:** Samiullah Khan

PONE-D-25-66618R1

PLOS One

Dear Dr. Alqahtani,

I'm pleased to inform you that your manuscript has been deemed suitable for publication in PLOS One. Congratulations! Your manuscript is now being handed over to our production team.

At this stage, our production department will prepare your paper for publication. This includes ensuring the following:

* All references, tables, and figures are properly cited

* All relevant supporting information is included in the manuscript submission,

* There are no issues that prevent the paper from being properly typeset

You will receive further instructions from the production team, including instructions on how to review your proof when it is ready. Please keep in mind that we are working through a large volume of accepted articles, so please give us a few days to review your paper and let you know the next and final steps.

Lastly, if your institution or institutions have a press office, please let them know about your upcoming paper now to help maximize its impact. If they'll be preparing press materials, please inform our press team within the next 48 hours. Your manuscript will remain under strict press embargo until 2 pm Eastern Time on the date of publication. For more information, please contact onepress@plos.org.

You will receive an invoice from PLOS for your publication fee after your manuscript has reached the completed accept phase. If you receive an email requesting payment before acceptance or for any other service, this may be a phishing scheme. Learn how to identify phishing emails and protect your accounts at https://explore.plos.org/phishing.

If we can help with anything else, please email us at customercare@plos.org.

Thank you for submitting your work to PLOS One and supporting open access.

Kind regards,

PLOS One Editorial Office Staff

on behalf of

Dr. Samiullah Khan

Academic Editor

PLOS One
